# High-Throughput Analysis of the Flagella FliK-Dependent Surfaceome and Secretome in *Bacillus thuringiensis*

**DOI:** 10.3390/biology14050525

**Published:** 2025-05-09

**Authors:** Carine Mouawad, Mireille Kallassy Awad, Carine Rodrigues-Machado, Céline Henry, Vincent Sanchis-Borja, Laure El Chamy

**Affiliations:** 1 Unité de Recherche Environnement, Génomique et Protéomique, Faculté des Sciences, Université Saint-Joseph de Beyrouth-Liban, Mar Roukos, Mkalles, Beirut 1107 2050, Lebanon; carine.mouawad@usj.edu.lb (C.M.); mireille.kallassy@usj.edu.lb (M.K.A.); 2 Université Paris-Saclay, INRAE, AgroParisTech, Micalis Institute, PAPPSO, 78350 Jouy-en-Josas, France; carine.rodrigues-machado@inrae.fr (C.R.-M.); celine.henry@inrae.fr (C.H.); 3 Université Paris-Saclay, INRAE, AgroParisTech, Micalis Institute, 78350 Jouy-en-Josas, France

**Keywords:** flagella, FliK, *Bacillus thuringiensis*, surfaceome, secretome, virulence, antimicrobial peptides resistance

## Abstract

Bacterial pathogens employ multiple strategies to invade and damage host tissues while evading immune defenses. Recent studies highlight flagella as crucial contributors to bacterial virulence, not only by facilitating motility, but also by regulating the secretion of virulence factors. However, the role of the flagella-dependent secretome remains largely unexplored. We have recently shown that FliK, a key regulator that defines substrate specificity in the flagellar export apparatus, is essential for the resistance of *Bacillus thuringiensis* (*B. thuringiensis*) against antimicrobial peptides (AMPs) and its virulence in a *Drosophila* infection model. In this study, we used liquid chromatography–tandem mass spectrometry to conduct a large-scale comparative analysis of the proteins secreted in culture supernatant or associated with the cell wall of the Δ*fliK* mutant and its reference strain. Our results reveal significant differences in the secretome and surfaceome of the Δ*fliK* mutant compared to the reference strain. These findings emphasize the role of FliK in regulating the production and secretion of several proteins, underscoring the importance of flagella in controlling various biological processes. This work provides valuable insights into the functional characterization of potential candidate proteins involved in *B. thuringiensis* virulence and AMP resistance mechanisms. Overall, these results open new perspectives for understanding the molecular processes that govern bacterial resistance to AMPs.

## Simple Summary

Bacteria employ diverse virulence strategies to invade host tissues and damage them while evading immune defenses. Growing evidence indicates that flagella structures, primarily involved in motility, also contribute to different aspects of virulence beyond movement/motility. In particular, the secretion apparatus of the flagellum, which governs its assembly, also plays a crucial role in the secretion of virulence factors. Using *Bacillus thuringiensis* (*B. thuringiensis*), a bacterium known for its use as a biocontrol agent, we have recently identified FliK, a key component of the flagella export apparatus, as essential for resistance to antimicrobial peptides, which act at the forefront of highly conserved immune defenses. To better understand the role of FliK, we conducted a large-scale comparative analysis of the protein composition secreted by a *fliK*-deficient strain and its reference counterpart. Our findings reveal that the absence of FliK and of a functional flagellar apparatus significantly alters the secreted protein profile of *B. thuringiensis*. Most importantly, our study identifies promising candidate proteins for further investigation, potentially unveiling new strategies to combat antibiotic resistance.

## 1. Introduction

Bacterial pathogens employ multiple strategies to invade and damage host tissues while subverting or eluding host defenses. Flagella have long been recognized as key contributors to virulence through motility-based functions, allowing bacteria to navigate towards the most favorable environments within the host. However, accumulating evidence suggests that flagella also play a prominent role in other stages of infections, including adhesion, biofilm formation, the modulation of host immune responses, and the secretion of virulence factors [1,2]. The flagellum is a complex self-assembling nanomachine that includes its own type III secretion system (T3SS), which is known for its role in the coordinated secretion of its external flagellar components [3]. Several studies have also pointed to the implication of the flagellar apparatus in the secretion of virulence factors in various bacterial species [1,4]. These findings indicate that the flagella T3SS serves as a conserved secretion apparatus influencing host–pathogen interaction. However, an in-depth understanding of flagella-dependent secretome remains largely unexplored.

The *Bacillus cereus* (*B. cereus*) group includes a growing number of Gram-positive spore-forming bacteria species with closely related phylogeny [5]. Four of these species are pathogenic: the anthrax agent *B. anthracis*, the foodborne pathogens *B. cereus sensu stricto* and *B. cytotoxicus*, and the entomopathogen *B. thuringiensis*, which is widely used as a biocontrol agent [5,6]. *B. cereus* species are ubiquitous in nature and their spores are resistant to common sterilization techniques, making them a major concern in the food industry. Although food poisoning with *B. cereus* is usually mild, it has been associated with serious infections in immunocompromised patients and preterm neonates, also leading to complications with extraintestinal infections such as septicemia, endocarditis, and vision-threatening endophthalmitis [7]. While the plasmid-borne genes determine the vulnerable hosts of the *B. cereus* group species, these bacteria share a common genetic background, including many genes linked to the expression of their virulence phenotypes [6]. In line with these findings, some *B. thuringiensis* strains have also been reported to cause infections in immunocompromised patients [8,9,10,11,12]. These data, combined with the increased spread of these bacteria in the environment, as a result of their growing use as biocontrol agents, emphasize the need for a thorough characterization of the genetic determinants of the *B. cereus* virulence phenotype.

We have previously reported that *B. cereus sensu stricto* and *B. thuringiensis* are highly resistant to antimicrobial peptides (AMPs), which serve as highly conserved key effectors at the front line of hosts’ innate immune defenses. This resistance largely explains their prominent virulence phenotype upon a septic injury infection in insect models such as *Galleria mellonella* and *Drosophila melanogaster* [13,14,15] and relies, among other factors, on the D-alanine esterification of teichoic acids through the activity of the gene products of the *dlt* operon, which has been described in several Gram-positive species [16,17,18,19,20,21,22]. To explore the novel genes required for the resistance of *B. thuringiensis* to cationic AMPs, we have recently performed a random mutagenesis of the acrystalliferous *Bt407* Cry- strain, which we screened in a two-step strategy combining in vitro and in vivo analysis. This study identified the *fliK* gene, which encodes a protein with a flagellar hook length control, as an essential determinant for *B. thuringiensis* resistance to AMPs and virulence in a *Drosophila* systemic infection model [23]. In particular, the *Bt* Δ*fliK* mutant is highly sensitive to polymyxin B and has an IC50 fourfold lower than that of *the reference strain*. Moreover, unlike its parental strain, which is highly virulent to both wild-type and AMP-deficient mutant flies, the *fliK* deletion mutant is only lethal to the latter. Interestingly, we also demonstrated that *B. thuringiensis* FliK-dependent resistance to AMPs is independent of its role in flagellar assembly and associated motility functions [23]. Indeed, the *Bt* Δ*fliK* mutant is non-flagellated and exhibits highly compromised motility and biofilm formation, consistent with the conserved function of FliK in other bacterial species [24,25,26,27,28,29,30,31]. However, comparative phenotypic analyses, including of the ∆*fla* deletion mutant, in which the genes encoding flagella proteins were deleted, show that only the ∆*fliK* mutant is sensitive to AMPs in vitro and in vivo. Notably, both ∆*fliK* and ∆*fla* mutants triggered an enhanced expression of AMP-encoding genes in infected flies. These data suggest that the structure or exposure of the peptidoglycan in bacteria lacking a flagella is somehow altered, leading to an enhanced sensing of the infection by the insect innate immune system. However, this does not explain the increased sensitivity of *Bt* ∆*fliK* to AMPs compared to the ∆*fla* mutant.

Although our data point out FliK as an essential element for the enhanced resistance of *B. thuringiensis* to AMPs, the molecular mechanism underlying this function remains to be clarified. In *B. subtilis*, FliK was shown to play an essential role in switching the substrate specificity of the flagellar export type III system by modifying the gate proteins FlhA and FlhB, which control the flagellar substrate’s export specificity [32,33,34,35,36,37,38]. When the hook reaches its mature length, this modification allows the export machinery to switch from rod-/hook-type proteins to filament-type proteins. This allows the termination of hook assembly and the initiation of filament formation [39,40,41,42]. In *B. subtilis*, this switch in export specificity also results in the secretion of FlgM, an anti-σ^D^ factor that regulates the transcription of the late flagellar gene [43,44,45,46,47,48,49,50]. In *B. thuringiensis*, the expression of some secreted virulence determinants was also shown to be dependent on a functional flagellar export apparatus [51,52,53,54]. Based on these findings, we sought to explore the flagella FliK-dependent secretome with particular emphasis on the secreted proteins that may account for *Bt407* virulence and its resistance to AMPs. To achieve this goal, we performed a large-scale comparative analysis of the proteins secreted in culture supernatant or those associated with the cell wall of the *Bt* Δ*fliK* mutant and its parental reference strain using liquid chromatography–tandem mass spectrometry (LC-MS/MS). The results we report here show significant variations in the secretome and the surfaceome compositions between the ∆*fliK* mutant and the reference strain. Most prominently, our data point to a marked reduction in virulence proteins in the secretome of ∆*fliK* and a noticeable increase in cell wall remodeling factors in its surfaceome. Differences also include proteins of unknown function as well as several proteins involved in cell division, proteolysis, stress response, and metabolic processes. These findings emphasize the role of FliK in regulating the production and/or secretion of multiple proteins, and underline the prominent role of the flagella in controlling various biological processes. Altogether, these variations may hold for the pleotropic phenotype of the ∆*fliK* mutant. Most importantly, our results provide a valuable list of candidates for further in-depth functional investigation to elucidate the mechanisms underlying the role of FliK in the resistance of *B. thuringiensis* to AMPs and its virulence.

## 2. Materials and Methods

### 2.1. Bacterial Strains and Growth Conditions

The acrystalliferous strain *Bacillus thuringiensis 407* Cry-(*Bt407* Cry-), originally derived from an environmental Cry+ serotype H1 strain 407 isolated in Brazil and rendered acrystalliferous by culturing at 42 °C [55], and the mutant strain *Bt407* Cry-Δ*fliK*, obtained through an in-frame deletion of the *fliK* gene via Splicing by Overlap Extension (SOE), as described in [23], were used throughout this study as the reference and mutant strains, respectively. An isolated colony of each strain, cultured on Luria–Bertani (LB) agar plates, was used to inoculate 4 mL of LB medium. This preculture was grown at 30 °C with agitation at 200 rpm until it reached an optical density at 600 nm (OD600) of 2 (~2 × 10^8^ bacteria/mL). A serial dilution ranging from 10^−1^ to 10^−5^ was performed in a final volume of 1 mL. The 10^−5^ dilution (~10^3^ bacteria/mL) was used to prepare cultures of dilutions 10^−6^, 10^−7^, and 10^−8^ in 10 mL of LB medium in a 100 mL Erlenmeyers. After 15 h of growth at 30 °C with shaking (200 rpm), one of the cultures, having reached the exponential growth phase (OD600 ~2 to 3), was used to inoculate 50 mL of LB medium in a 500 mL Erlenmeyer, to obtain an initial OD600 of 0.2. This protocol enabled us to optimize bacterial growth to obtain 50 mL cultures at the exponential growth phase (OD600 = 2) in less than 3 h of incubation. This protocol was repeated three times for the two bacterial strains.

### 2.2. Sample Preparation for LC-MS/MS Analysis of Secreted Proteins in Culture Supernatant

Supernatants of 3 independent 50 mL cultures of *Bt407* Cry- and *Bt* Δ*fliK*, grown in LB broth to the exponential phase (OD600 = 2), were collected via centrifugation for 10 min at 3500 g and filtered on Millipore membranes with a porosity 0.22 μm and stored at −20 °C until protein digestion. Sample preparation for secretome analysis was performed using the PAPPSO platform. The supernatants were concentrated via ultrafiltration using an Amicon Ultra centrifugal filters UF 3 kDa filter at 12,000 g for 40 min at 4 °C. After drying using a SpeedVac, the concentrated supernatants were denaturated in a LDS (Lithium Dodecyl Sulfate) sample buffer at 95 °C for 15 min then loaded on SDS-PAGE gel. The proteins were in-gel digested with trypsin, according to the protocol described in [56]. Briefly, the gels were washed with (i) 10% acetic acid and 40% ethanol and then (ii) with 100 μL of washing buffer containing acetonitrile/ammonium bicarbonate in a 1:1 proportion for 15 min. They were subsequently dehydrated with 100 μL of 100% acetonitrile. Reduction was performed using 50 μL of the fresh 10 mM dithiothreitol at 56 °C for 30 min. Alkylation was performed using 50 μL of freshly prepared 55 mM iodoacetamide for 45 min at room temperature in the dark. The gels were then rinsed with acetonitrile/ammonium bicarbonate in a 1:1 proportion for 15 min and dried with 100% acetonitrile. The gel pieces were rehydrated on ice with 100 ng of trypsin and digestion was performed overnight at 37 °C. After adding 10 μL of 50 mM ammonium bicarbonate for 10 min, peptides were extracted by incubating gel pieces in extraction solvent (0.5% trifluoroacetic acid/50% acetonitrile) for 15 min, and transferred into new tubes. The gels were dried with 100 μL of 100% acetonitrile, and the supernatants were transferred into the previous tubes. The peptides were finally dried in a SpeedVac. The dried extract peptides were dissolved in 20 μL of loading buffer (98% H_2_O, 2% acetonitrile, and 0,08% trifluoroacetic acid) just before mass spectrometry analysis.

### 2.3. Sample Preparation for LC-MS/MS Analysis of Cell-Wall-Associated Protein

Pellets obtained from 50 mL cultures of *Bt407* Cry- and *Bt* Δ*fliK* (OD = 2) were suspended in 5 mL of washing buffer (PBS1X + Sucrose 40%, pH 7.4). One mL of this buffer was first added to gently resuspend the pellet, and then the remaining volume was added. After 10 min of centrifugation at 3500 g and 4 °C, the pellets were gently resuspended in 5 mL of digestion buffer (PBS1X + Sucrose 40% + 1 mM CaCl2 pH 7.4). Samples were digested by adding 1 μg of trypsin (Sequencing Grade Modified Trypsin—Promega) to a 1.5 mL volume of each bacterial suspension for 5 min at 37 °C. This protocol was set up to allow for limited cell lysis, as confirmed by comparable CFU counts of the microbial pellets obtained with or without trypsin treatment (Supplementary Table S1) and the absence of nucleic acid in the hydrolysate supernatants, attested by agarose gel electrophoresis (Supplementary Figure S1). The supernatants of the *Bt407* Cry- and the *Bt* Δ*fliK* strains (in biological triplicates) obtained after trypsin digestion were filtered on Millipore membranes with porosity of 0.22 μm and subsequently stored at −20 °C until proteomic analysis. Trypsin-untreated suspensions served as a negative control for each bacterial strain throughout the procedure. Sample preparation for shaving analysis was performed using the PAPPSO platform. The samples were purified and desalted in solid-phase extraction using a polymeric C18 column. The peptides were eluted with 70% acetonitrile and 0,1% trifluoroacetic acid and dried using SpeedVac. They were suspended in 20 μL of loading buffer (98% H_2_O, 2% acetonitrile, and 0,08% trifluoroacetic acid) and diluted 1/14 just before mass spectrometry analysis.

### 2.4. LC-MS/MS Analysis and Protein Identification

Mass spectrometry was performed using the PAPPSO platform (MICALIS, INRAE, Jouy-en-Josas, France; http://pappso.inrae.fr/ (accessed on 7 May 2025)) using an Orbitrap Fusion^TM^ Lumos^TM^ TribridT^M^ (Thermo Fisher Scientific, San Diego, CA, USA) coupled to an UltiMate^TM^ 3000 RSLC nanoLC System (Thermo Fisher Scientific). The tryptic peptides were loaded on a PepMap Neo trap column (300 μm i.d. × 5 mm, with a particle size of 5 μm, 100 Å, Thermo Fisher), and were separated using a C18 column (50 cm × 75 μm i.d. 2 μm particle size, Thermo Fisher Scientific, San Diego, CA, USA). The peptides were eluted on the nanoLC system through the following gradient elution program: 2.5–35% buffer B (80% acetonitrile and 0.1% formic acid) within 0–50 min, 35–45% buffer B in 50–55 min, and 45–98% buffer B in 55–57 min. The detected peptides were acquired in the DDA mode. For MS1 signals, the electrospray voltage was set at 1600 V, the temperature of the ion transfer tube at 275 °C, and the MS1 Orbitrap resolution at 120,000 (at *m*/*z* 200), with the standard gain control (AGC) target and maximum injection time of 100 ms. For MS/MS signals, the MS/MS Orbitrap resolution and AGC depended on the expected total of peptides in the samples. The MS/MS isolation window was set at 1.6 Da, standard AGC target, the dynamic exclusion time at 60 s, Orbitrap resolution at 30,000, and the dynamic maximum injection time mode and higher energy collisional dissociation (HCD) with collision energy at 30%. The data were converted into an mzXML format using MS convert (ProteoWizard, version 3.0.8934). Protein identification and filtering were performed by querying MS/MS data against the *Bacillus thuringiensis* 407 database (NCBI_Bacillus_thuringiensis407cry_6402entries_18102022.fasta) together with a custom contaminant database (trypsin and keratins), using X!Tandem Alanine (2017.2.1.4; [57]) and i2MassChroQ software (version 0.4.72) developed by the PAPPSO facility ([58], http://pappso.inrae.fr/bioinfo/ (accessed on 7 May 2025)). The identified proteins were filtered with a minimum of two different peptides, with a peptide E-value < 10^−2^ and protein E value < 10^−4^.

### 2.5. Protein Quantification and Statistical Analysis of LC-MS/MS Data

Relative quantification of protein abundances was performed using two complementary methods: spectral counting (SC), defined as the number of MS2 spectra assigned to a protein [59], and eXtracted Ion Chromatograms (XICs), defined as the sum of the MS1 intensities of all peptides associated with a protein [60].

Different bioinformatic pipelines were applied as indicated. For SC, this involved (i) the removal of proteins having < five spectra in all samples, and (ii) the removal of proteins showing an abundance variation < 1.5 between strains. For XICs, it included (i) the removal of peptides with a high retention time variation > 30 s and peak width > 200 s, (ii) the normalization of peptide intensities based on a reference sample, (iii) the removal of peptides with >5% of missing values in the whole experiment, (iv) the removal of shared peptides, (v) the peptides correlated to a reference peptide being kept for further analysis, (vi) the missing values of peptide intensities being imputed by replacing them with the minimum abundance obtained for this protein in the whole experiment, (vii) the removal of proteins quantified with low peptide number (<2), and (viii) the removal of proteins showing an abundance variation < 1.5 between strains.

The protein abundance changes were detected via ANOVA tests for the SC and XIC methods. The abundance of a protein was considered significantly variable when the adjusted *p* value was < 0.05. Finally, the non-cytoplasmic proteins, which were considered statistically significantly variable (at padj values < 0.05) between *Bt407* Cry- and *Bt* Δ*fliK*, were kept by using the online Psort database (www.psort.org (accessed on 7 May 2025), version 3.0.3).

## 3. Results and Discussion

### 3.1. Global Proteomic Analysis of Cell Surface and Secreted Proteins in the Bt407 ΔfliK Mutant and Its Reference Strain

Gram-positive bacteria resist cationic AMPs through different mechanisms, including cell wall modifications and alterations in the cell membrane composition that reduce AMPs’ attraction to target membranes. Additional resistance strategies involve the sequestration, inhibition, or degradation of AMPs via surface or secreted proteins [61,62]. Based on these data, we decided to investigate the role of FliK in regulating the soluble and/or cell-surface associated proteins essential for AMP resistance in the strain *Bt407* by conducting a global proteomic analysis. For that, we harvested three independent bacterial cultures of the reference strain *Bt407* Cry- and the *Bt* ∆*fliK* strains in the exponential phase to collect supernatants containing soluble protein candidates. The bacterial pellets were subsequently treated with trypsin to collect cell-surface-attached target candidate proteins. This protocol was set up to limit bacterial lysis, as confirmed by CFU counting before and after trypsin treatment and by the absence of DNA in the recovered cell supernatant (see material and methods Supplementary Table S1 and Supplementary Figure S1) [63]. Throughout the experiment, trypsin-untreated pellets served as negative controls for both the reference strain and the *Bt* Δ*fliK* mutant. The collected secretome and surfaceome of the *Bt* Δ*fliK* mutant were analyzed both qualitatively and quantitatively, relative to the reference strain, using LC-MS/MS.

The combined data of the surfaceome reference and mutant samples identified 491 protein subgroups, corresponding to 4938 distinct peptides, while 640 proteins subgroups, corresponding to 8059 distinct peptides, were identified from the secretome samples. The False Discovery Rates (FDRs) for peptides and proteins were estimated to be 0.08% and 0.06%, and 0.04% and 0.03%, for the surfaceome and secretome, respectively, confirming analytical reliability. Importantly, almost no peptides were detected in the trypsin-untreated pellets for both the reference strain and the *Bt* Δ*fliK* mutant, indicating that protein shedding was very limited in our experimental conditions and confirming the accuracy of our surfaceome analysis strategy. The detected peptides were grouped into proteins sharing at least one common peptide and subgroups of proteins having identical peptide sets (Supplementary Table S2).

**Figure 1:**
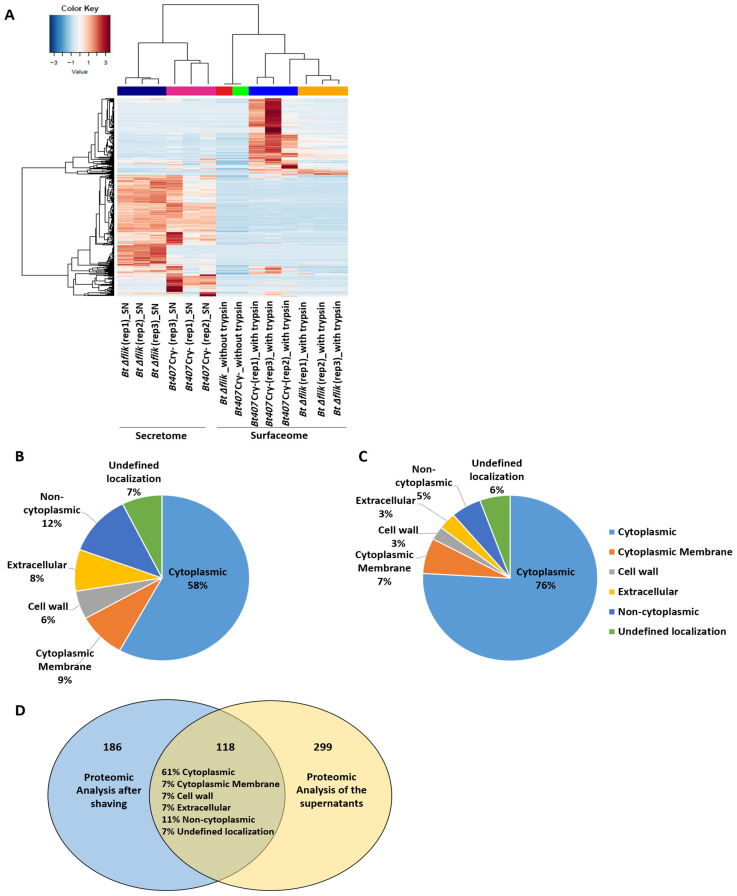
Overall approach to proteomic analysis. (**A**) Heatmap representation, obtained from spectral count analysis, of secreted and cell surface proteins from *Bt407* Cry- and *Bt* Δ*fliK* with a reliable number of spectra (≥5) in each sample. (**B**) Distribution of the subcellular localization, obtained using the online database PSORTb, version 3.0.3, of the reliable proteins identified from the proteomic analysis of the supernatant. (**C**) Distribution of the subcellular localization, obtained using the online database PSORTb, version 3.0.3, of the reliable proteins identified from the proteomic analysis after shaving. (**D**) Venn diagram depicting the number of reliable proteins identified in each proteomic analysis in both analyses, as well as an estimate of the percentage of subcellular localization for the proteins identified in both analyses.

A global relative quantification analysis was first performed using the spectral count (SC) strategy to compare the data retrieved from all samples. A heatmap representation of all subgroups of proteins detected by more than five spectra was generated (Figure 1A). Although there was some heterogeneity among the *Bt407* biological replicates, this representation clearly separated the secretome and surfaceome samples, highlighting a distinct subset of soluble-secreted and surface-associated proteins.

To better assess the data retrieved from each proteomic study, we used the online database PSORTb, version 3.0.3 (www.psort.org (accessed on 7 May 2025)) [64] to predict for the subcellular localization of the identified proteins (Figure 1B,C). According to this database, 119 of the 5520 proteins encoded in the *B. thuringiensis* genome are known to be secreted, and 63 are known to be associated with the cell wall. Our study detected 34.5% of the secreted proteins, representing approximately 6% of the total proteins identified in the secretome samples, and 23.8% of the cell wall-associated proteins in *B. thuringiensis*, representing approximately 3% of the total proteins identified in the surfaceome samples. This online prediction tool also indicated that 58% of the proteins found in the culture supernatants of *Bt407* were cytoplasmic proteins (Figure 1B). These results are consistent with previous findings, which showed a similar proportion of cytoplasmic proteins in the secretome of *B. cereus* [65]. This percentage rises to 76% for the proteins identified in our surfaceome samples (Figure 1C). Similar results have been previously observed for several Gram-positive bacteria, including *B. subtilis* [66,67]. For the latter, it was previously reported that about half of the extracellular proteins are not predicted to be secreted [68]. Several hypotheses have been advanced to explain the presence of predicted cytoplasmic proteins in the secretomes and surfaceomes of bacteria. Among these are cell lysis, release within membrane vesicles, and the activity of yet unidentified export pathways capable of translocating proteins lacking known secretion/exporting or retention motifs [63,66,67]. It is predicted that such proteins, referred to as anchorless proteins, may attach to the surfaceome via non-covalent electrostatic interactions with negatively charged molecules like teichoic acids. While the role of these proteins remains under investigation, universally conserved cytoplasmic proteins are believed to help bacteria evade detection by the host immune system. Anchorless proteins can be divided into low-affinity binders that are easily shed from the cell, and high-affinity binders that can only be detected upon proteolytic cleavage [67]. Our data indicate that cytoplasmic proteins account for 61% of the proteins shared by the secretome and surfaceome of *Bt407*, suggesting that these proteins could be low-affinity binders found in both fractions of our proteomic analysis. Our data also point out that our trypsin treatment resulted in a low-rate cell lysis that was not detected by our CFU counts or nucleic acid detection controls. In these conditions, the high sensitivity of mass spectrometry would allow for the detection of intracellular proteins, even if present in small proportions. Nevertheless, secreted and cell-wall-associated proteins remain highly detectable in the secretome and surfaceome analyses, confirming the effectiveness of our proteomic approach.

### 3.2. Comparative Analysis of the Surfaceomes of the Bt ΔfliK and Reference Bt407 Cry- Strains

**Figure 2:**
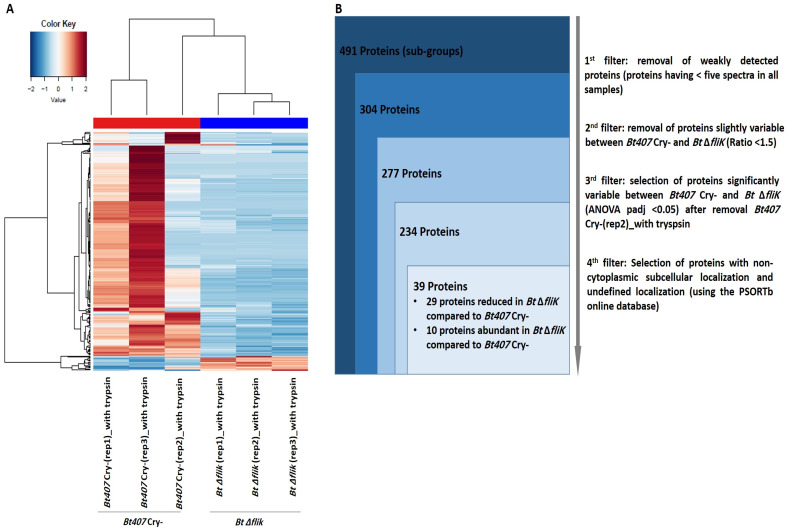
Proteomic analysis performed on spectral count data after shaving of *Bt407* Cry- and *Bt* Δ*fliK*. (**A**) Heatmap representation of proteins obtained from spectral count data analysis after shaving of *Bt407* Cry- and *Bt* Δ*fliK*, showing a reliable number of spectra (≥5) in the six shaved samples and exhibiting a variability of more than 1.5 between the two conditions. (**B**) Filtration flow was carried out on the 491 proteins to select non-cytoplasmic proteins that exhibited significant variability between *Bt407* Cry- and *Bt* Δ*fliK*.

Next, we looked for the qualitative and quantitative differences in SC found between the surfaceome of *Bt* ∆*fliK* and that of its reference parental strain. Due to the heterogeneity observed among *Bt407* Cry- replicates in our initial global analysis (Figure 1A), we refined our approach by repeating the heatmap analysis on the surfaceome data, by filtering for proteins with a variation threshold > 1.5 between the reference and the mutant strains. The data shown in Figure 2A confirm the variations among *Bt407* Cry- replicates. To better assess this variability among the samples, we performed a principal component analysis (PCA). The data shown in Figure S2 confirm that the shaved replicates of the mutant strain were well grouped, while the reference strain replicates were more dispersed, with the sample “*Bt407* Cry-(rep2)_with trypsin” being particularly eccentric. Therefore, we decided to exclude this sample from the analysis. Out of 277 proteins, 234 showed significant variations, with at least a 1.5-fold change in relative abundance (padj < 0.05) of spectra count between the reference and the mutant strain, with 39 predicted to be non-cytoplasmic or of undefined locations (Figure 2B). Of these, 29 were significantly reduced in *Bt* ∆*fliK* compared to in the reference strain (Table 1), while 10 showed an inverse pattern (Table 2).

**Figure 3:**
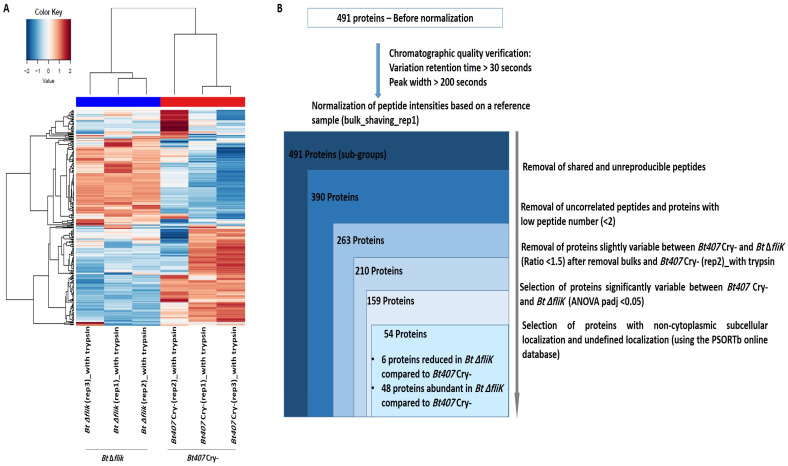
Proteomic analysis performed on XIC data after shaving of *Bt407* Cry- and *Bt* Δ*fliK*. (**A**) Heatmap representation of proteins of the surfaceome obtained from XIC data analysis and containing specific, reproducible, and correlated peptides, quantified with a minimum of two peptides. (**B**) Filtration flow carried out on the 491 proteins for the selection of non-cytoplasmic proteins that exhibited significant variability between *Bt407* Cry- and *Bt* Δ*fliK*.

In a complementary approach, the data retrieved from the LC-MS/MS were analyzed using the eXtracted Ion Chromatogram (XIC) strategy. Upon the removal of unreproducible and uncorrelated peptides, as well as proteins with low peptide numbers (<2), the data retrieved for 263 proteins were included in a heatmap analysis. The results confirmed the previously observed divergence between *Bt407* Cry- replicates (Figure 3A). Consistent with SC data, PCA performed on XIC data indicated that the sample “*Bt407* Cry-(rep2)_with trypsin” is eccentric to the other two biological replicates that grouped together (Figure S3). This sample was then also excluded from the analysis of XIC data. From 159 proteins that differed significantly between *Bt407* Cry- and *Bt* Δ*fliK*, 54 were non-cytoplasmic or had undefined localization and showed at least a 1.5-fold change in relative abundance (padj < 0.05) (Figure 3B). Six of these proteins showed decreased abundance in the ∆*fliK* mutant compared to the reference strain and were also previously found using the SC strategy (Table 3). The remaining 48 proteins, including the 10 candidates previously identified using the SC strategy, showed increased abundance in the surfaceome of *Bt* ∆*fliK* (Table 4).

In agreement with FliK’s established role in switching the substrate specificity of the flagellar secretion apparatus, our analysis revealed that the *Bt* ∆*fliK* mutant contains distinct amounts of rod and filament proteins on its surface compared to the reference strain. Specifically, the *Bt* ∆*fliK* mutant contained higher amounts of the flagellar basal body rod protein FlgC, while showing decreased amounts of flagellin B, a filament-type protein, on its surface compared to the reference strain.

Based on the assigned or predicted functions verified using the KEGG (https://www.genome.jp/kegg/ (accessed on 7 May 2025)), UniProt (https://www.uniprot.org/ (accessed on 7 May 2025)), InterPro (https://www.ebi.ac.uk/interpro/ (accessed on 7 May 2025)), and NCBI databases, the proteins retrieved via our comparative proteomic analysis are involved in diverse biological processes (Table 1, Table 2, Table 3 and Table 4).

In particular, we found that several proteins involved in stress response, metabolic processes’ cell division, proteolysis, and protein export, along with some proteins of an unknown function, were significantly reduced in the surfaceome of the Δ*fliK* mutant compared to the reference strain (Table 1 and Table 3), likely contributing to its pleiotropic phenotype. Notably, the tyrosine protein kinase YwqD and the GTP-binding protein TypA were absent or present in trace amounts in the Δ*fliK* surfaceome (Table 1). YwqD belongs to a protein family that is involved in the assembly and export of complex polysaccharides, which are key components of biofilms [69], suggesting its role in biofilm formation. Likewise, the GTP-binding protein TypA, also known as BipA, a member of the superfamily of ribosome-binding GTPases within the TRAFAC class (translation factors) of GTPases [70,71,72], was present only in trace amounts in the surfaceome of the Δ*fliK* mutant compared to the reference strain (Table 1). Although its precise function is still poorly understood, TypA/BipA is thought to regulate virulence and stress responses in different bacteria [73,74,75], including *P. aeruginosa* PAO1, where it was associated with swarming motility and biofilm formation [76]. Importantly, in 2013, Neidig et al. demonstrated that a *typA* mutant in *P. aeruginosa* PA14 was attenuated in rapid cell surface attachment, displayed reduced biofilm formation, and exhibited an increased antibiotic sensitivity to ß-lactam, tetracycline, and antimicrobial peptide (Polymixin B). In addition, this mutation resulted in the reduced virulence of *P. aeruginosa* PA14 and caused the down-regulation of important virulence-related genes, such as those involved in the regulation and assembly of the type III secretion system [77]. Drawing parallels with *P. aeruginosa*, further investigation is needed to determine whether the loss of TypA/BipA correlates with impaired biofilm formation and AMP sensitivity in *B. thuringiensis*.

Our analysis also revealed a significant 1.5-fold increase in the amount of secreted proteins in the surfaceome of the Δ*fliK* mutant. Prominent among these are proteins involved in cell wall remodeling and cell adhesion and membrane-damaging proteins. This finding is consistent with a previous study conducted on the Δ*secDF* mutant, which exhibited reduced cellular flagellation and motility and an up-regulated cell wall stress response [65]. This enrichment of the enzymes responsible for cell turnover aligns with the increased immuno-stimulatory potential of the Δ*fliK* mutant in the *Drosophila* model compared to the reference strain, likely due to enhanced peptidoglycan release or the presence of specific peptidoglycan immuno-stimulatory fragments that activate AMP production in vivo [23]. However, while the *Bt* Δ*fla* mutant exhibits a similar immuno-stimulatory potential to the *Bt* Δ*fliK* mutant strain, it is more virulent than the Δ*fliK* mutant in *Drosophila*, correlating with the latter’s increased sensitivity to AMPs both in vitro and in vivo. This result underscores that cell wall perturbations alone do not fully explain sensitivity differences to AMPs, warranting further comparative studies between Δ*fla* and Δ*fliK* mutants to fully understand the role and relative contributions of cell-wall-related elements in these phenotypic differences.

### 3.3. Comparative Analysis of the Secretomes of the Bt ΔfliK and Reference Bt407 Cry- Strains

**Figure 4:**
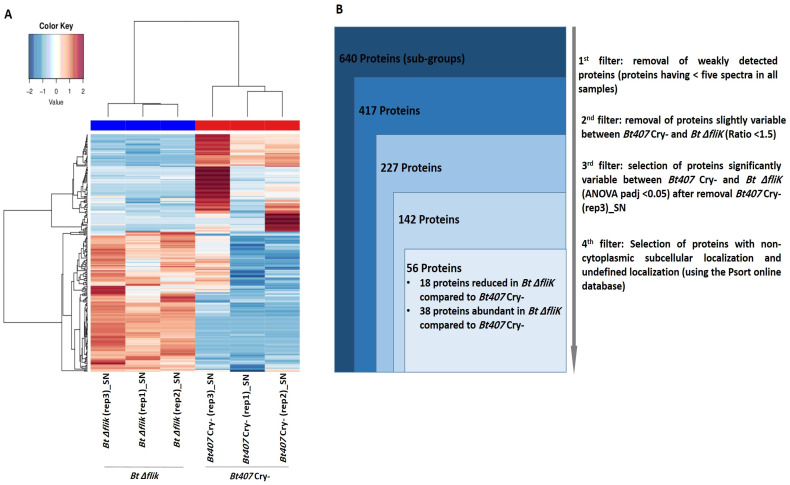
Proteomic analysis performed on spectral count data from the supernatants of *Bt407* Cry- and *Bt* Δ*fliK*. (**A**) Heatmap representation of proteins obtained through spectral count data analysis of the secretome of *Bt407* Cry- and *Bt* Δ*fliK*, showing a reliable number of spectra (≥5) in the six supernatant samples and exhibiting a variability of more than 1.5 between the two conditions. (**B**) Filtration flow carried out on the 640 proteins to select non-cytoplasmic proteins that exhibited significant variability between *Bt407* Cry- and *Bt* Δ*fliK*.

**Figure 5:**
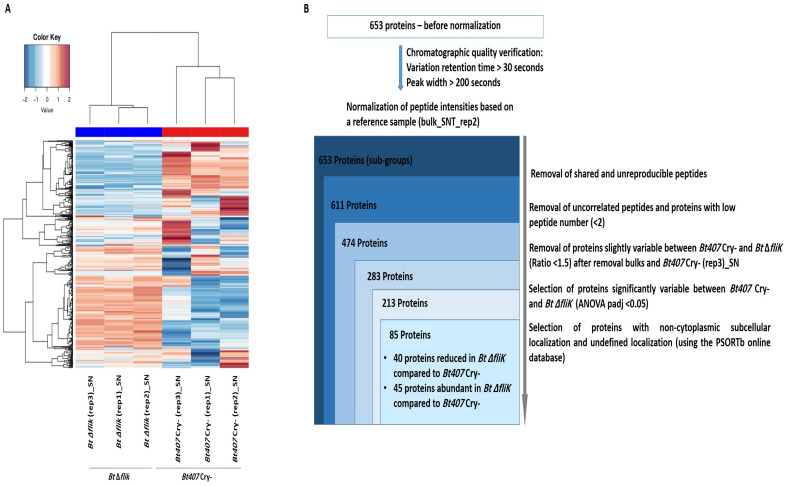
Proteomic analysis performed on XIC data from the supernatants of *Bt407* Cry- and *Bt* Δ*fliK*. (**A**) Heatmap representation of proteins of the secretome obtained through XIC data analysis of *Bt407* Cry- and *Bt* Δ*fliK* containing specific, reproducible, and correlated peptides, and quantified with a minimum of two peptides. (**B**) Filtration flow carried out to select non-cytoplasmic proteins that exhibited significant variability between *Bt407* Cry- and *Bt* Δ*fliK*.

We then analyzed the differences between the secretomes of ∆*fliK* and of its parental strain using the SC strategy. A heatmap analysis, including proteins significantly detected in the secretome with a number of spectra ≥ 5 and a variation threshold > 1.5 between the reference and the mutant strain, is presented in Figure 4A. *Bt* ∆*fliK* replicates grouped, while the previously observed divergence between *Bt407* Cry- replicates persisted, even using the filter newly applied to data analysis. This was further confirmed by PCA, which indicated one eccentric sample (*Bt407* Cry-(rep3)_SN) relative to the two other biological replicates (Figure S4). Similar results were obtained from secretome data analysis using the XIC strategy (Figure 5A and Figure S5). Consequently, the *Bt407* Cry-(rep3)_SN sample was excluded from the analysis. Out of 227 proteins, 142 showed significant variations in SC between the reference and the mutant strains (Figure 4B), with 56 proteins predicted to be non-cytoplasmic or of an undefined location. Of these, 18 showed reduced amounts and 38 increased amounts, with at least a 1.5-fold change in relative abundance (padj < 0.05) in *Bt* ∆*fliK* compared to the reference strain (Table 5 and Table 6). The XIC method confirmed these results, identifying 85 proteins that differed significantly between *Bt* Δ*fliK and Bt407* Cry-, with non-cytoplasmic or undefined subcellular localization (Figure 5B). Approximately half of these were less abundant in the mutant strain (Table 7), whereas the other half showed increased amounts compared to the reference strain (Table 8).

As in the surfaceome analysis, our results showed a significant reduction in filament-type substrates (FlgK, FlgL, and FliD) and concurrent increases in rod–hook substrates (FlgC and FlgE) in the Δ*fliK* mutant’s secretome, further validating our experimental procedure (Table 5, Table 6, Table 7 and Table 8) [36,37,38,78]. Indeed, due to the frequent flagellar turnover, flagellum components are documented to be commonly found in the bacterial secretomes [65,79,80,81]. As expected, the flagellar hook length control protein BTB_c16930 “FliK” was absent in the *Bt* Δ*fliK* mutant secretome. Furthermore, its presence in the secretome of the reference strain is consistent with previous data obtained from a study performed on the secretome of *Salmonella* [82]. Interestingly, we observed decreased amounts of FlgE in the ∆*fliK* mutant surfaceome but increased amounts in its secretome compared to the reference strain. These findings may be relevant to the occurrence of elongated hook structures, so-called polyhooks, in *fliK* loss-of-function mutants of *B. subtilis* ([30] and Ole Andreas Okstad personal communication). Beyond flagellar components, multiple proteins significantly affected in the secretome of the *Bt* ∆*fliK* mutant were involved in diverse processes, including possible virulence mechanisms, metabolic processes, cell adhesion, and cell envelope composition (Table 5, Table 6, Table 7 and Table 8). Notably, our results indicate an approximate two-fold reduction in the levels of the components of the enterotoxins HBL-L1 and Nhe-L2 (NheA) in the supernatant of the ∆*fliK* mutant compared to the reference strain (Table 7), consistent with previous studies showing a reduction in the HBL components in the supernatant of the ∆*flhA* mutant [51,53]. Several other putative virulence factors similarly decreased in the supernatant of the ∆*fliK* mutant (Table 5 and Table 7), potentially explaining the ∆*fliK* mutant’s reduced cytoxicity against epithelial human cells compared to the reference *Bt407* strain (Attieh Zaynoun personal communication). While reporter gene assays have indicated that *flhA* regulates the transcription of the *hbl* genes, HBL, Nhe, and CytK enterotoxin secretion has been shown to be dependent on the Sec translocation pathway [51,52]. Interestingly, our data indicate an approximately 5.5-fold accumulation of HBL-L1 on the surface of the *Bt* ∆*fliK* mutant compared to the reference strain (Table 4) alongside microbial collagenase (ColA), which was significantly reduced in the secretome of ∆*fliK*, but accumulated in the surfaceome (Table 4 and Table 7). These findings suggest a complex and intricate FliK-dependent mechanism governing the secretion of virulence factors from the cell surface into its surrounding environment, once addressed to the cell surface. This further emphasizes a long-surmised coordination between bacterial motility and the secretion of virulence determinants [51,52,53], although the regulation mechanism remains unclear. Interestingly, we have also observed a complete absence of “oxidoreductase, short chain dehydrogenase/reductase family superfamily”, “putative thiol peroxidase Tpx”, and “uncharacterized protein YjlC” from the surfaceome of the *Bt* Δ*fliK* mutant (Table 1), but these proteins were present at 2 to 3-fold higher levels in its secretome compared to *Bt407* Cry- (Table 6 and Table 8). However, a clear understanding of the role of these proteins in the observed phenotypes of *Bt* Δ*fliK* will require further investigation into their specific involvement and regulation. Similarly, and in line with our surfaceome analysis, data retrieved from the secretome analysis clearly indicate a 1.5 to 3-fold increase in the amount of proteins involved in cell wall turnover (Table 8).

Several other proteins identified in our analysis may contribute to the ∆*fliK* mutant’s increased AMP sensitivity. Notably, we observed a significant reduction, by at least 50%, in the levels of serine protease, a member of the subtilase family, in both the secretome and surfaceome of the Δ*fliK* mutant compared to the reference strain (Table 1, Table 5 and Table 7). This protein belongs to the S8 peptidase family and shares approximately 99% similarity with a putative collagenase identified in the secretome of *B. cereus* [65], suggesting a possible role in *B. thuringiensis* virulence.

Among the proteins involved in metabolism and transport, we identified an “uncharacterized protein”, YkgB, and an “oligopeptide-binding protein”, OppA, both previously linked to antibiotic resistance. Whilst SC analysis detected these proteins exclusively in the secretome of the *Bt* Δ*fliK* mutant (Table 6), XIC analysis revealed that the OppA levels were doubled in the secretome of the mutant compared to the reference strain (Table 8). According to the KEGG database, the uncharacterized protein YkgB is a 6-phosphogluconolactonase (6-pgl). Pgl is an enzyme in the pentose phosphate pathway that converts 6-phosphogluconolactone into 6-phosphogluconate. In *S. aureus*, the *pgl* mutant had a thick cell wall, a ruffled cell surface, and exhibited high resistance to β-lactam antibiotic and reduced lipoteichoic acid (LTA) levels, leading to a significantly increased positive surface charge [83]. Since a well-established bacterial resistance strategy for positively charged AMPs often involves reducing the cell surface’s negative charge, to limit electrostatic interaction with AMPs, further research should be conducted to investigate the impact of increased 6-pgl abundance in the Δ*fliK* mutant on AMP sensitivity. OppA, the substrate-binding protein of the Opp system (ATP-binding cassette transporter), was also significantly enriched in the secretome of the *Bt* Δ*fliK* mutant. OppA was found to play a role in the uptake of antibiotics in *E. coli*, where decreased *oppA* gene expression was associated with aminoglycoside antibiotic resistance [84]. While its potential role in AMP uptake remains untested in *B. thuringiensis*, investigating whether increased OppA levels in the Δ*fliK* mutant contribute to its sensitivity to AMPs could yield important insights.

## 4. Conclusions

Previous studies have focused on analyzing the entire proteome and/or the secretome of *B. cereus* and *B. thuringiensis*, but to our knowledge, no studies have previously explored the surfaceomes of these bacteria species at a high-throughput level [65,79,80,85,86,87,88,89]. Our study is novel in its dual comparative analysis of the surfaceomes and the secretomes of both the reference and ∆*fliK* mutant strains, with the goal of understanding the role of FliK and, by extension, that of the flagellar apparatus in the regulation of secreted proteins in *B. thuringiensis*. While Bouillaut et al. [51] previously investigated this question by conducting a two-dimensional electrophoresis analysis of the reference and ∆*flhA* mutant supernatants [51], our combined SC and XIC quantification methods identified 29 and 45 proteins with reduced abundance and 48 and 64 proteins with increased abundance, at the cell surface and in the secretome, respectively, of the ∆*fliK* mutant compared to the reference *Bt407* strain. This integrated analysis of surfaceomes and secretomes provides novel insights into FliK’s crucial role in regulating the cell surface and secreted proteins involved in various biological processes. Our findings reveal not only FliK’s direct influence on flagellar components’ distribution, but also its broader impact on virulence factors’ secretion and localization, although it remains unclear whether this regulation occurs at the transcriptional, translational, or secretion level. Importantly, our comparative proteomic analysis provides a list of promising candidates for in-depth functional analysis to totally elucidate the mechanisms by which FliK contributes to *B. thuringiensis* virulence and resistance to AMPs. Complementary transcriptomic analyses would be useful to fully elucidate FliK’s regulatory functions and identify potential novel targets for the development of new antibacterial therapeutic strategies.

## Figures and Tables

**Table 1:** List of proteins with reduced abundance in *Bt* Δ*fliK* compared to *Bt407* Cry- based on spectral count analysis of the surfaceome. Proteins were assigned to a biological process and annotated using KEGG [1], UniProt [2], InterPro [3], and Ncbi [4] databases. Proteins highlighted in red were also detected during XIC quantification of the surfaceome analysis. The subcellular localization prediction was performed using the online database PSORTb, version 3.0.3.

SC Quantification					
Biological Process	Proteins Reduced in *Bt ΔfliK* Compared to *Bt407* Cry-	Localization Prediction	Ratio *Bt* Δ*fliK*/ *Bt407* Cry-	Padj	Databases
Flagellum assembly	AFV17395.1 flagellin B	Undefined	0.38	2.51 × 10^−10^	[1]
	AFV17389.1 flagellar hook protein FlgE	Extracellular	0.15	1.02 × 10^−3^	[1]
Proteolysis	AFV19517.1 serine protease, subtilase family	Non-cytoplasmic	0.56	3.06 × 10^−2^	[2,3]
	AFV19362.1 protease HhoA	Undefined	0.22	5.68 × 10^−3^	[2,3]
Stress response	AFV19771.1 GTP-binding protein TypA	Cytoplasmic Membrane	0.04	7.79 × 10^−7^	[4]
Metabolic process	AFV16964.1 enoyl-[acyl-carrier-protein] reductase FabI	Cytoplasmic Membrane	0.09	5.34 × 10^−13^	[1,2,3]
	AFV16923.1 3-oxoacyl-[acyl-carrier-protein] synthase 2	Cytoplasmic Membrane	0.00	3.60 × 10^−21^	[1,2,3]
	AFV16410.1 sphingomyelinase C	Extracellular	0.39	1.40 × 10^−3^	[1,2]
Extracellular polysaccharide biosynthetic process	AFV21128.1 tyrosine protein kinase YwqD	Cytoplasmic Membrane	0.00	7.86 × 10^−5^	[3]
Regulation of cell division	AFV20267.1 septum site-determining protein MinD	Cytoplasmic Membrane	0.03	9.93 × 10^−10^	[3,4]
	AFV20522.1 DNA translocase SftA	Cytoplasmic Membrane	0.00	3.28 × 10^−5^	[1,2]
Protein export	AFV19653.1 signal recognition particle protein Ffh	Cytoplasmic Membrane	0.00	2.32 × 10^−6^	[1,2,3]
Unclassified	AFV20645.1 phage shock protein A	Cytoplasmic Membrane	0.32	1.75 × 10^−4^	-
	AFV16253.1 putative cytosolic protein	Undefined	0.09	3.06 × 10^−8^	-
	AFV17720.1 oxidoreductase, short-chain dehydrogenase/reductase family superfamily	Non-cytoplasmic	0.00	1.94 × 10^−4^	-
	AFV20471.1 putative thiol peroxidase Tpx	Undefined	0.00	3.25 × 10^−14^	-
	AFV20858.1 FeS cluster assembly protein SufD	Undefined	0.09	5.77 × 10^−5^	-
	AFV20943.1 uncharacterized protein YjlC	Undefined	0.00	7.66 × 10^−11^	-
Hypothetical proteins	AFV20367.1 hypothetical protein BTB_c46880	Extracellular	0.20	7.38 × 10^−5^	-
	AFV15824.1 hypothetical protein BTB_c00190	Undefined	0.24	1.07 × 10^−2^	-
	AFV17737.1 hypothetical protein BTB_c20450	Non-cytoplasmic	0.14	9.93 × 10^−10^	-
Proteins carried by the plasmid 502	AFV22050.1 hypothetical protein BTB_502p07450 (plasmid)	Undefined	0.56	2.71 × 10^−3^	-
	AFV21751.1 hypothetical protein BTB_502p04460 (plasmid)	Undefined	0.19	9.97 × 10^−5^	-
	AFV21840.1 hypothetical protein BTB_502p05350 (plasmid)	Undefined	0.57	1.66 × 10^−2^	-
	AFV21949.1 hypothetical protein BTB_502p06440 (plasmid)	Cytoplasmic Membrane	0.55	3.39 × 10^−2^	-
	AFV21845.1 hypothetical protein BTB_502p05400 (plasmid)	Undefined	0.27	4.58 × 10^−3^	-
	AFV22055.1 hypothetical protein BTB_502p07500 (plasmid)	Undefined	0.14	4.54 × 10^−6^	-
	AFV22023.1 hypothetical protein BTB_502p07180 (plasmid)	Undefined	0.00	9.91 × 10^−7^	-
	AFV21509.1 hypothetical protein BTB_502p02040 (plasmid)	Non-cytoplasmic	0.29	8.28 × 10^−3^	-

**Table 2:** List of proteins with increased abundance in *Bt* Δ*fliK* compared to *Bt407* Cry- based on spectral count analysis of the surfaceome. Proteins were assigned to a biological process and annotated using KEGG [1], UniProt [2], and InterPro [3] databases. Proteins highlighted in red were also detected in XIC quantification analysis of the surfaceome. The subcellular localization prediction was performed using the online database PSORTb, version 3.0.3.

SC Quantification					
Biological Process	Proteins Abundant in *Bt ΔfliK* Compared to *Bt407* Cry-	Localization Prediction	Ratio *Bt* Δ*fliK*/ *Bt407* Cry-	Padj	Databases
Cell wall turnover	AFV21084.1 cell wall-binding protein YocH	Non-cytoplasmic	Absent	4.46 × 10^−6^	[3]
	AFV16578.1 cell wall-binding protein YocH	Undefined	2.86	5.01 × 10^−15^	[2,3]
	AFV17714.1 endopeptidase LytF (lytF1)	Non-cytoplasmic	1.66	3.94 × 10^−3^	[1]
Cell adhesion	AFV21209.1 LPXTG-motif cell wall anchor domain protein	Cell wall	3.53	5.44 × 10^−5^	[3]
Membrane-damaging toxins	AFV20963.1 hemolysin	Extracellular	8.00	9.68 × 10^−3^	[1]
Transmembrane transport	AFV16934.1 dipeptide-binding protein DppE	Cell wall	Absent	1.42 × 10^−4^	[1,3]
Unclassified	AFV20752.1 cell surface protein	Cell wall	Absent	4.46 × 10^−6^	-
	AFV18997.1 surface protein, LPXTG-motif cell wall anchor domain protein	Cell wall	Absent	2.74 × 10^−10^	-
Hypothetical proteins	AFV17929.1 hypothetical protein BTB_c22370	Non-cytoplasmic	7.33	6.25 × 10^−4^	-
	AFV19452.1 hypothetical protein BTB_c37700	Non-cytoplasmic	2.07	3.84 × 10^−2^	-

**Table 3:** List of proteins with reduced abundance in *Bt* Δ*fliK* compared to *Bt407* Cry- based on XIC analysis of the surfaceome. Proteins highlighted in red were also detected during spectral count quantification analysis of the surfaceome. The subcellular localization prediction was performed using the online database PSORTb, version 3.0.3.

XIC Quantification				
Biological Process	Proteins Reduced in *Bt ΔfliK* Compared to *Bt407* Cry-	Localization Prediction	Ratio *Bt* Δ*fliK*/ *Bt407* Cry-	Padj
Unclassified	AFV20645.1 phage shock protein A	Cytoplasmic Membrane	0.32	1.26 × 10^−2^
	AFV16253.1 putative cytosolic protein	Undefined	0.27	1.80 × 10^−2^
Hypothetical proteins	AFV20367.1 hypothetical protein BTB_c46880	Extracellular	0.53	5.34 × 10^−3^
	AFV15824.1 hypothetical protein BTB_c00190	Undefined	0.57	7.14 × 10^−3^
Hypothetical proteins carried by the plasmid 502	AFV22050.1 hypothetical protein BTB_502p07450 (plasmid)	Undefined	0.59	1.39 × 10^−2^
	AFV21751.1 hypothetical protein BTB_502p04460 (plasmid)	Undefined	0.51	3.98 × 10^−2^

**Table 4:** List of proteins with increased abundance in *Bt* Δ*fliK* compared to *Bt407* Cry- based on XIC analysis of the surfaceome. Proteins were assigned to a biological process and annotated using KEGG [1], UniProt [2], and InterPro [3] databases. Proteins highlighted in red were also detected in spectral count quantification analysis of the surfaceome. The subcellular localization prediction was performed using the online database PSORTb, version 3.0.3.

XIC Quantification						
Biological Process		Proteins Abundant in *Bt ΔfliK* Compared to *Bt407* Cry-	Localization Prediction	Ratio *Bt* Δ*fliK/Bt407* Cry-	Padj	Databases
Flagellum assembly		AFV17380.1 flagellar basal body rod protein FlgC	Undefined	5.01	1.88 × 10^−2^	[1]
Cell adhesion		AFV21209.1 LPXTG-motif cell wall anchor domain protein	Cell wall	30.16	4.57 × 10^−3^	[3]
Cell wall turnover		AFV21084.1 cell wall-binding protein YocH	Non-cytoplasmic	54.51	8.42 × 10^−4^	[3]
		AFV16578.1 cell wall-binding protein YocH	Undefined	5.50	3.81 × 10^−2^	[2,3]
		AFV17714.1 endopeptidase LytF (lytF1)	Non-cytoplasmic	8.36	5.34 × 10^−3^	[1]
		AFV21114.1 transcriptional regulator LytR	Cytoplasmic Membrane	7.10	1.37 × 10^−2^	[1,2]
		AFV18765.1 cell wall-binding protein YocH	Non-cytoplasmic	6.11	5.48 × 10^−3^	[3]
		AFV16473.1 cell wall-binding protein YocH	Non-cytoplasmic	5.42	1.37 × 10^−2^	[2,3]
		AFV21077.1 endopeptidase LytF (lytf3)	Non-cytoplasmic	11.02	1.79 × 10^−3^	[1]
		AFV16653.1 N-acetylmuramoyl-L-alanine amidase CwlH	Cell wall	8.79	5.80 × 10^−3^	[3]
		AFV17283.1 penicillin-binding protein 1A/1B	Cytoplasmic Membrane	4.80	1.29 × 10^−2^	[1]
		AFV20098.1 uncharacterized protein YqgF	Cytoplasmic Membrane	3.91	3.25 × 10^−2^	[1,3]
		AFV19021.1 N-acetylmuramoyl-L-alanine amidase XlyA	Cell wall	3.73	8.17 × 10^−3^	[3]
		AFV21075.1 lipoteichoic acid synthase-like YqgS	Cytoplasmic Membrane	5.89	1.19 × 10^−2^	[1]
Bacterial toxins	Membrane-damaging toxins	AFV20963.1 hemolysin	Extracellular	16.59	7.87 × 10^−3^	[1]
		AFV18241.1 hemolysin BL-binding component HblA	Extracellular	12.01	9.94 × 10^−3^	[1]
		AFV18240.1 hemolysin BL lytic component L1	Extracellular	5.68	4.05 × 10^−2^	[1]
		AFV16857.1 gamma-hemolysin component B	Extracellular	5.64	1.18 × 10^−2^	[1]
		AFV18239.1 hemolysin BL lytic component L2	Non-cytoplasmic	5.24	3.40 × 10^−3^	[1]
		AFV17552.1 hemolysin BL-binding component HblA	Extracellular	4.53	1.40 × 10^−2^	[1]
	Extracellular matrix-damaging toxins	AFV16287.1 microbial collagenase ColA	Extracellular	5.34	7.14 × 10^−3^	[1]
Transmembrane transport		AFV16934.1 dipeptide-binding protein DppE	Cell wall	8.60	4.57 × 10^−3^	[1,3]
		AFV19546.1 putative lipoprotein YufN	Non-cytoplasmic	5.67	9.94 × 10^−3^	[1]
		AFV21101.1 putative efflux system component YknX	Non-cytoplasmic	2.92	1.00 × 10^−2^	[1]
Proteolysis		AFV15871.1 ATP-dependent zinc metalloprotease FtsH	Cytoplasmic Membrane	7.28	6.92 × 10^−3^	[3]
		AFV16405.1 immune inhibitor A	Extracellular	3.10	3.66 × 10^−2^	[2,3]
Metabolic process		AFV18894.1 endonuclease YhcR	Cell wall	4.03	1.18 × 10^−2^	[1,3]
		AFV18743.1 putative polysaccharide deacetylase YheN	Non-cytoplasmic	6.11	1.61 × 10^−2^	[3]
Unclassified		AFV20752.1 cell surface protein	Cell wall	5.98	1.35 × 10^−2^	-
		AFV18997.1 surface protein, LPXTG-motif cell wall anchor domain protein	Cell wall	4.04	7.04 × 10^−3^	-
		AFV19048.1 LPXTG-motif cell wall anchor domain protein	Cell wall	4.82	1.27 × 10^−2^	-
		AFV16010.1 invasion protein IagB domain protein	Cytoplasmic Membrane	6.27	9.94 × 10^−3^	-
		AFV19026.1 PGA biosynthesis protein CapA	Cytoplasmic Membrane	5.23	8.17 × 10^−3^	-
Hypothetical proteins		AFV17929.1 hypothetical protein BTB_c22370	Non-cytoplasmic	7.86	3.00 × 10^−3^	-
		AFV17420.1 hypothetical protein BTB_c17260	Undefined	4.76	1.27 × 10^−2^	-
		AFV21218.1 hypothetical protein BTB_c55680	Non-cytoplasmic	4.82	1.39 × 10^−2^	-
		AFV19452.1 hypothetical protein BTB_c37700	Non-cytoplasmic	4.28	1.39 × 10^−2^	-
		AFV16704.1 hypothetical protein BTB_c10090	Extracellular	3.29	9.94 × 10^−3^	-
		AFV20642.1 hypothetical protein BTB_c49630	Cytoplasmic Membrane	3.02	2.99 × 10^−2^	-
		AFV21086.1 hypothetical protein BTB_c54360	Undefined	2.98	1.41 × 10^−2^	-
Proteins carried by the plasmid 502 and the plasmid 78		AFV21822.1 hypothetical protein BTB_502p05170 (plasmid)	Extracellular	8.50	1.87 × 10^−2^	-
		AFV21352.1 hypothetical protein BTB_502p00160 (plasmid)	Undefined	4.57	1.45 × 10^−2^	-
		AFV21846.1 hypothetical protein BTB_502p05410 (plasmid)	Non-cytoplasmic	3.93	1.28 × 10^−2^	-
		AFV21957.1 hypothetical protein BTB_502p06520 (plasmid)	Extracellular	3.75	3.06 × 10^−2^	-
		AFV21370.1 hypothetical protein BTB_502p00340 (plasmid)	Non-cytoplasmic	3.24	4.80 × 10^−2^	-
		AFV21847.1 penicillin-binding protein 1A (plasmid)	Cytoplasmic Membrane	2.71	3.60 × 10^−2^	-
		AFV22044.1 hypothetical protein BTB_502p07390 (plasmid)	Undefined	1.95	3.89 × 10^−2^	-
		AFV22124.1 hypothetical protein BTB_78p00520 (plasmid)	Cell wall	5.91	5.34 × 10^−3^	-

**Table 5:** List of proteins with reduced abundance in *Bt* Δ*fliK* compared to *Bt407* Cry- based on spectral count analysis of the secretome. Proteins were assigned to a biological process and annotated using KEGG [1], UniProt [2], InterPro [3], and Ncbi [4], databases. Proteins highlighted in red were also detected in XIC quantification of the secretome analysis. The subcellular localization prediction was performed using the online database PSORTb, version 3.0.3.

SC Quantification					
Biological Process	Proteins Reduced in *Bt ΔfliK* Compared to *Bt407* Cry-	Localization Prediction	Ratio *Bt ΔfliK*/ *Bt407* Cry-	Padj	Databases
Flagellum assembly	AFV17376.1 flagellar hook-associated FliD	Undefined	0.04	7.71 × 10^−59^	[1]
	AFV17374.1 flagellar hook-associated protein FlgK	Undefined	0.00	1.07 × 10^−43^	[1]
	AFV17387.1 flagellar hook length control protein BTB_c16930 FliK	Undefined	0.00	4.77 × 10^−13^	[1]
	AFV17375.1 flagellar hook-associated protein 3 FlgL	Undefined	0.23	3.06 × 10^−8^	[1]
Membrane-damaging toxins	AFV16409.1 phospholipase C	Extracellular	0.73	3.78 × 10^−2^	[1]
Proteolysis	AFV19517.1 serine protease, subtilase family	Non-cytoplasmic	0.48	4.65 × 10^−6^	[2,3]
Unclassified	AFV19126.1 viral-enhancing factor	Undefined	0.56	5.77 × 10^−3^	-
Proteins carried by the plasmid 502 and the plasmid 78	AFV22044.1 hypothetical protein BTB_502p07390 (plasmid)	Undefined	0.69	3.30 × 10^−4^	-
	AFV21505.1 hypothetical protein BTB_502p02000 (plasmid)	Non-cytoplasmic	0.52	6.42 × 10^−5^	-
	AFV21509.1 hypothetical protein BTB_502p02040 (plasmid)	Non-cytoplasmic	0.58	5.50 × 10^−3^	-
	AFV22021.1 hypothetical protein BTB_502p07160 (plasmid)	Non-cytoplasmic	0.00	7.24 × 10^−4^	-
	AFV21504.1 hypothetical protein BTB_502p01990 (plasmid)	Non-cytoplasmic	0.31	5.74 × 10^−4^	-
	AFV21503.1 TPR-repeat-containing protein (plasmid)	Non-cytoplasmic	0.49	4.52 × 10^−6^	-
	AFV21501.1 TPR-repeat-containing protein (plasmid)	Non-cytoplasmic	0.38	4.35 × 10^−7^	-
	AFV21502.1 TPR-repeat-containing protein (plasmid)	Non-cytoplasmic	0.14	2.26 × 10^−7^	-
	AFV21908.1 TROVE domain-containing protein (plasmid)	Cytoplasmic Membrane	0.04	2.12 × 10^−11^	-
	AFV21346.1 single-stranded DNA-binding protein (plasmid)	Undefined	0.13	1.75 × 10^−4^	-
	AFV22123.1 conjugation protein (plasmid)	Non-cytoplasmic	0.24	4.98 × 10^−4^	-

**Table 6:** List of proteins with increased abundance in *Bt* Δ*fliK* compared to *Bt407* Cry- quantified through spectral count analysis of the secretome. Proteins were assigned to a biological process and annotated using KEGG [1], UniProt [2], and InterPro [3] databases. Proteins highlighted in red were also detected in XIC quantification of the secretome analysis. The subcellular localization prediction was performed using the online database PSORTb, version 3.0.3.

SC Quantification					
Biological Process	Proteins Abundant in *Bt ΔfliK* Compared to *Bt407* Cry-	Localization Prediction	Ratio *Bt ΔfliK*/ *Bt407* Cry-	Padj	Databases
Flagellum assembly	AFV17389.1 flagellar hook protein FlgE	Extracellular	1.53	2.33 × 10^−3^	[1]
Metabolic process	AFV19113.1 uncharacterized protein YkgB	Undefined	Absent	5.74 × 10^−4^	[1]
	AFV17736.1 putative polysaccharide deacetylase YheN	Non-cytoplasmic	2.53	7.60 × 10^−3^	[2,3]
	AFV20423.1 malate dehydrogenase Mdh	Non-cytoplasmic	6.33	2.93 × 10^−5^	[1,2,3]
	AFV21296.1 superoxide dismutase sodA	Extracellular	Absent	8.17 × 10^−8^	[3]
	AFV18629.1 GlcNAc-binding protein A	Non-cytoplasmic	Absent	2.23 × 10^−4^	[1]
	AFV19919.1 endonuclease YhcR	Cell wall	6.00	1.30 × 10^−7^	[1,2,3]
	AFV20972.1 carboxylesterase Est	Cytoplasmic Membrane	5.33	1.02 × 10^−2^	[1]
Antibiotic catabolic process	AFV18265.1 beta-lactamase Bla	Extracellular	1.96	1.19 × 10^−2^	[1,3]
	AFV18892.1 D-alanyl-D-alanine carboxypeptidase	Extracellular	Absent	4.21 × 10^−3^	[1]
	AFV19308.1 D-alanyl-D-alanine carboxypeptidase	Cytoplasmic Membrane	2.44	4.93 × 10^−2^	[3]
Transmembrane transport	AFV16390.1 oligopeptide-binding protein OppA	Cell wall	Absent	5.74 × 10^−4^	[1,2,3]
	AFV16928.1 dipeptide-binding protein DppE	Cell wall	Absent	1.58 × 10^−3^	[1,2,3]
Cell adhesion	AFV21209.1 LPXTG-motif cell wall anchor domain protein	Cell wall	2.48	2.75 × 10^−9^	[3]
	AFV16822.1 collagen adhesion protein	Undefined	7.33	1.93 × 10^−2^	[3]
Cell redox homeostasis	AFV20748.1 ferredoxin--NADP reductase	Cytoplasmic Membrane	10.00	3.33 × 10^−5^	[3]
Dephosphorylation	AFV20169.1 alkaline phosphatase 3	Cytoplasmic Membrane	Absent	8.17 × 10^−8^	[3]
Proteolysis	AFV17022.1 immune inhibitor A	Extracellular	2.76	1.83 × 10^−5^	[3]
	AFV20917.1 neutral protease B	Extracellular	Absent	1.13 × 10^−16^	[3]
	AFV16332.1 bacillolysin	Extracellular	6.00	5.25 × 10^−4^	[2,3]
	AFV20341.1 putative carboxypeptidase YodJ	Cytoplasmic Membrane	2.48	2.12 × 10^−3^	[2,3]
Cell wall turnover	AFV16578.1 cell wall-binding protein YocH	Undefined	1.66	5.35 × 10^−6^	[2,3]
	AFV19469.1 cell wall-binding protein YocH	Non-cytoplasmic	2.74	7.50 × 10^−5^	[2]
Unclassified	AFV20752.1 cell surface protein	Cell wall	3.11	2.93 × 10^−5^	-
	AFV20609.1 cell surface protein	Non-cytoplasmic	Absent	1.58 × 10^−3^	-
	AFV17720.1 oxidoreductase, short-chain dehydrogenase/reductase family superfamily	Non-cytoplasmic	6.67	2.94 × 10^−2^	-
	AFV20529.1 putative aminopeptidase YtoP	Undefined	10.00	3.33 × 10^−5^	-
	AFV19291.1 vancomycin B-type resistance protein	Non-cytoplasmic	3.20	1.77 × 10^−5^	-
	AFV20774.1 phage protein	Undefined	Absent	7.38 × 10^−11^	-
	AFV16435.1 prophage antirepressor	Undefined	Absent	1.47 × 10^−5^	-
Hypothetical proteins	AFV16451.1 hypothetical protein BTB_c07330	Undefined	Absent	1.88 × 10^−18^	-
	AFV20938.1 hypothetical protein BTB_c52870	Non-cytoplasmic	Absent	3.93 × 10^−13^	-
	AFV20606.1 hypothetical protein BTB_c49270	Extracellular	Absent	7.23 × 10^−10^	-
	AFV20607.1 hypothetical protein BTB_c49280	Non-cytoplasmic	Absent	2.30 × 10^−5^	-
	AFV19994.1 hypothetical protein BTB_c43120	Non-cytoplasmic	4.17	3.39 × 10^−3^	-
	AFV16908.1 hypothetical protein BTB_c12130	Undefined	14.67	1.55 × 10^−4^	-
	AFV19008.1 hypothetical protein BTB_c33240	Non-cytoplasmic	2.27	2.21 × 10^−2^	-
Proteins carried by the plasmid 502 and the plasmid 78	AFV21623.1 hypothetical protein BTB_502p03180 (plasmid)	Cytoplasmic Membrane	2.86	9.55 × 10^−3^	-

**Table 7:** List of proteins with reduced abundance in *Bt* Δ*fliK* compared to *Bt407* Cry- based on XIC analysis of the secretome. Proteins were assigned to a biological process and annotated using KEGG [1], UniProt [2], and InterPro [3] databases. Proteins highlighted in red were also detected in spectral count quantification analysis of the secretome. The subcellular localization prediction was performed using the online database PSORTb, version 3.0.3.

XIC Quantification						
Biological Process		Proteins Reduced in *Bt ΔfliK* Compared to *Bt407* Cry-	Localization Prediction	Ratio *Bt ΔfliK*/ *Bt407* Cry-	Padj	Databases
Flagellum assembly		AFV17374.1 flagellar hook-associated protein FlgK	Undefined	0.07	3.17 × 10^−2^	[1]
		AFV17376.1 flagellar hook-associated FliD	Undefined	0.08	2.28 × 10^−2^	[1]
		AFV17387.1 flagellar hook length control protein BTB_c16930 FliK	Undefined	0.34	4.77 × 10^−3^	[1]
Proteolysis		AFV19517.1 serine protease, subtilase family	Non-cytoplasmic	0.21	2.35 × 10^−3^	[2,3]
Bacterial toxins	Membrane-damaging toxins	AFV16409.1 phospholipase C	Extracellular	0.38	3.26 × 10^−3^	[1]
		AFV18240.1 hemolysin BL lytic component L1	Extracellular	0.64	1.19 × 10^−2^	[1]
		AFV17551.1 Non-hemolytic enterotoxin lytic component L2	Non-cytoplasmic	0.63	4.17 × 10^−2^	[1]
		AFV16857.1 gamma-hemolysin component B	Extracellular	0.42	6.17 × 10^−3^	[1]
	Extracellular matrix-damaging toxins	AFV16287.1 microbial collagenase ColA	Extracellular	0.39	1.25 × 10^−2^	[1]
Cell wall turnover		AFV16668.1 S-layer protein/N-acetylmuramoyl-L-alanine amidase	Cell wall	0.65	9.49 × 10^−3^	[3]
Unclassified		AFV16733.1 S-layer protein/peptidoglycan endo-beta-N-acetylglucosaminidase	Cell wall	0.56	2.18 × 10^−2^	-
		AFV17754.1 putative murein endopeptidase	Non-cytoplasmic	0.54	4.01 × 10^−2^	-
		AFV21260.1 FMN-dependent NADH-azoreductase	Undefined	0.21	1.25 × 10^−3^	-
		AFV21027.1 cell division ATP-binding protein FtsE	Cytoplasmic Membrane	0.45	7.65 × 10^−3^	-
		AFV19126.1 viral-enhancing factor	Undefined	0.47	1.82 × 10^−2^	-
		AFV19880.1 uncharacterized protein YpuA	Non-cytoplasmic	0.55	5.82 × 10^−3^	-
		AFV19518.1 cell wall hydrolase CwlJ	Extracellular	0.62	8.89 × 10^−3^	-
Hypothetical protein		AFV19684.1 hypothetical protein BTB_c40020	Non-cytoplasmic	0.39	2.99 × 10^−2^	-
Proteins carried by the plasmid 502 and the plasmid 78		AFV22044.1 hypothetical protein BTB_502p07390 (plasmid)	Undefined	0.40	6.19 × 10^−3^	-
		AFV21505.1 hypothetical protein BTB_502p02000 (plasmid)	Non-cytoplasmic	0.32	2.35 × 10^−3^	-
		AFV21509.1 hypothetical protein BTB_502p02040 (plasmid)	Non-cytoplasmic	0.40	2.35 × 10^−3^	-
		AFV22043.1 hypothetical protein BTB_502p07380 (plasmid)	Non-cytoplasmic	0.44	3.67 × 10^−2^	-
		AFV21504.1 hypothetical protein BTB_502p01990 (plasmid)	Non-cytoplasmic	0.34	8.76 × 10^−3^	-
		AFV21874.1 hypothetical protein BTB_502p05690 (plasmid)	Undefined	0.32	1.08 × 10^−2^	-
		AFV21389.1 hypothetical protein BTB_502p00530 (plasmid)	Non-cytoplasmic	0.49	1.72 × 10^−2^	-
		AFV22050.1 hypothetical protein BTB_502p07450 (plasmid)	Undefined	0.25	1.36 × 10^−2^	-
		AFV22055.1 hypothetical protein BTB_502p07500 (plasmid)	Undefined	0.42	3.95 × 10^−2^	-
		AFV22068.1 hypothetical protein BTB_502p07630 (plasmid)	Cytoplasmic Membrane	0.23	2.23 × 10^−2^	-
		AFV22066.1 hypothetical protein BTB_502p07610 (plasmid)	Undefined	0.19	1.36 × 10^−2^	-
		AFV21503.1 TPR-repeat-containing protein (plasmid)	Non-cytoplasmic	0.60	2.33 × 10^−2^	-
		AFV21501.1 TPR-repeat-containing protein (plasmid)	Non-cytoplasmic	0.35	1.25 × 10^−3^	-
		AFV21346.1 single-stranded DNA-binding protein (plasmid)	Undefined	0.12	4.15 × 10^−3^	-
		AFV22042.1 hypothetical protein BTB_502p07370 (plasmid)	Non-cytoplasmic	0.63	5.52 × 10^−3^	-
		AFV21809.1 hypothetical protein BTB_502p05040 (plasmid)	Undefined	0.60	7.42 × 10^−3^	-
		AFV21822.1 hypothetical protein BTB_502p05170 (plasmid)	Extracellular	0.49	5.57 × 10^−3^	-
		AFV21341.1 hypothetical protein BTB_502p00050 (plasmid)	Cell wall	0.40	1.11 × 10^−2^	-
		AFV21896.1 hypothetical protein BTB_502p05910 (plasmid)	Undefined	0.30	4.15 × 10^−3^	-
		AFV21606.1 hypothetical protein BTB_502p03010 (plasmid)	Undefined	0.15	1.72 × 10^−2^	-
		AFV21378.1 sporulation-specific N-acetylmuramoyl-L-alanine amidase (plasmid)	Extracellular	0.54	1.79 × 10^−3^	-
		AFV22124.1 hypothetical protein BTB_78p00520 (plasmid)	Cell wall	0.52	2.64 × 10^−3^	-

**Table 8:** List of proteins with increased abundance in *Bt* Δ*fliK* compared to *Bt407* Cry- based on XIC analysis of the secretome. Proteins were assigned to a biological process and annotated using KEGG [1], UniProt [2], and InterPro [3] databases. Proteins highlighted in red were also detected in spectral count quantification analysis of the secretome. The subcellular localization prediction was performed using the online database PSORTb, version 3.0.3.

XIC Quantification					
Biological Process	Proteins Abundant in *Bt ΔfliK* Compared to *Bt407* Cry-	Localization Prediction	Ratio *Bt ΔfliK*/ *Bt407* Cry-	Padj	Databases
Flagellum assembly	AFV17380.1 flagellar basal body rod protein FlgC	Undefined	1.97	2.83 × 10^−2^	[1]
Transmembrane transport	AFV16390.1 oligopeptide-binding protein OppA	Cell wall	2.15	3.85 × 10^−2^	[1,2,3]
Cell adhesion	AFV21209.1 LPXTG-motif cell wall anchor domain protein	Cell wall	2.06	1.79 × 10^−2^	[3]
	AFV16822.1 collagen adhesion protein	Undefined	3.96	7.07 × 10^−3^	[3]
Metabolic process	AFV17736.1 putative polysaccharide deacetylase YheN	Non-cytoplasmic	2.25	7.55 × 10^−3^	[2,3]
	AFV18743.1 putative polysaccharide deacetylase YheN	Non-cytoplasmic	1.59	7.56 × 10^−3^	[3]
	AFV19270.1 cellulase	Undefined	2.46	8.57 × 10^−3^	[1,3]
	AFV18629.1 GlcNAc-binding protein A	Non-cytoplasmic	1.95	2.64 × 10^−3^	[1]
	AFV18894.1 endonuclease YhcR	Cell wall	2.92	2.64 × 10^−3^	[1,3]
	AFV20100.1 superoxide dismutase SodA	Extracellular	2.67	5.90 × 10^−3^	[3]
	AFV20944.1 NADH dehydrogenase-like protein YjlD	Cytoplasmic Membrane	1.99	2.16 × 10^−2^	[1]
	AFV18210.1 putative agmatine deiminase AguA	Non-cytoplasmic	1.64	3.24 × 10^−3^	[1,3]
Antibiotic catabolic process	AFV18265.1 beta-lactamase Bla	Extracellular	1.91	2.23 × 10^−2^	[1,3]
Cell redox homeostasis	AFV20748.1 ferredoxin--NADP reductase	Cytoplasmic Membrane	3.59	2.35 × 10^−3^	[3]
Proteolysis	AFV18532.1 bacillolysin	Extracellular	2.14	1.79 × 10^−3^	[2,3]
	AFV16332.1 bacillolysin	Extracellular	2.53	2.49 × 10^−3^	[2,3]
	AFV20341.1 putative carboxypeptidase YodJ	Cytoplasmic Membrane	1.87	1.79 × 10^−3^	[2,3]
	AFV18874.1 signal peptidase I	Cell wall	1.92	2.03 × 10^−2^	[3]
Extracellular matrix-damaging toxins	AFV19160.1 collagenase	Extracellular	1.74	2.85 × 10^−2^	[1]
Cell wall turnover	AFV16578.1 cell wall-binding protein YocH	Undefined	2.21	3.63 × 10^−3^	[2,3]
	AFV21084.1 cell wall-binding protein YocH	Non-cytoplasmic	1.87	3.66 × 10^−3^	[3]
	AFV16473.1 cell wall-binding protein YocH	Non-cytoplasmic	1.68	2.64 × 10^−3^	[2,3]
	AFV18765.1 cell wall-binding protein YocH	Non-cytoplasmic	1.64	2.39 × 10^−2^	[3]
	AFV19520.1 lipoteichoic acid synthase-like YqgS	Cytoplasmic Membrane	1.76	2.66 × 10^−2^	[1]
Unclassified	AFV20752.1 cell surface protein	Cell wall	1.62	4.97 × 10^−2^	-
	AFV18997.1 surface protein, LPXTG-motif cell wall anchor domain protein	Cell wall	1.51	1.91 × 10^−2^	-
	AFV17720.1 oxidoreductase, short-chain dehydrogenase/reductase family superfamily	Non-cytoplasmic	3.08	2.85 × 10^−3^	-
	AFV20529.1 putative aminopeptidase YtoP	Undefined	2.79	1.62 × 10^−2^	-
	AFV20471.1 putative thiol peroxidase Tpx	Undefined	2.74	2.35 × 10^−3^	-
	AFV17554.1 O-GlcNAcase NagJ	Extracellular	2.01	2.35 × 10^−3^	-
	AFV15863.1 heat shock protein 15	Undefined	2.00	1.19 × 10^−2^	-
	AFV19291.1 vancomycin B-type resistance protein	Non-cytoplasmic	2.67	3.42 × 10^−3^	-
	AFV19051.1 extracellular ribonuclease Bsn	Extracellular	1.50	2.66 × 10^−2^	-
	AFV20943.1 uncharacterized protein YjlC	Undefined	1.81	4.97 × 10^−2^	-
	AFV16460.1 hypothetical protein BTB_c07420	Non-cytoplasmic	2.33	1.68 × 10^−2^	-
	AFV18460.1 hypothetical protein BTB_c27760	Non-cytoplasmic	2.03	3.79 × 10^−2^	-
	AFV19281.1 hypothetical protein BTB_c35990	Non-cytoplasmic	1.75	1.86 × 10^−2^	-
	AFV18355.1 hypothetical protein BTB_c26710	Cytoplasmic Membrane	1.61	1.66 × 10^−2^	-
	AFV18356.1 hypothetical protein BTB_c26720	Cytoplasmic Membrane	1.59	1.84 × 10^−2^	-
Hypothetical proteins	AFV19008.1 hypothetical protein BTB_c33240	Non-cytoplasmic	1.52	1.34 × 10^−2^	-
	AFV16451.1 hypothetical protein BTB_c07330	Undefined	1.94	3.01 × 10^−2^	-
	AFV19994.1 hypothetical protein BTB_c43120	Non-cytoplasmic	1.58	3.95 × 10^−2^	-
	AFV16908.1 hypothetical protein BTB_c12130	Undefined	2.66	1.07 × 10^−2^	-
Proteins carried by the plasmid 502 and the plasmid 78	AFV21623.1 hypothetical protein BTB_502p03180 (plasmid)	Cytoplasmic Membrane	2.70	7.55 × 10^−3^	-
	AFV22140.1 hypothetical protein BTB_78p00680 (plasmid)	Non-cytoplasmic	2.10	5.06 × 10^−3^	-

## Data Availability

The original contributions presented in this study are included in the article and Supplementary Materials. Further inquiries can be directed to the corresponding authors.

## Supplementary Materials

The following supporting information can be downloaded at https://www.mdpi.com/article/10.3390/biology14050525/s1. Figure S1: Evaluation of bacterial lysis by checking the presence/absence of nucleic acid on agarose gel. Absence of nucleic acid in the triplicates of the reference strain *Bt407* Cry-(A) and the mutant strain *Bt* Δ*fliK* (B) without or with treatment with 1 μg of trypsin for 5 min at 37 °C; Figure S2: Principal component analysis (PCA) performed on the proteins obtained from spectral count data after shaving of *Bt407* Cry- and *Bt* Δ*fliK*. PCA is represented by axes 1–2. *Bt407* Cry-_with trypsin in red and *Bt* Δ*fliK*_with trypsin in blue; Figure S3: Principal component analysis (PCA) performed on proteins obtained from XIC data after shaving of *Bt407* Cry- and *Bt* Δ*fliK*. PCA is represented by axes 1–2. *Bt407* Cry-_with trypsin in red, *Bt* Δ*fliK*_with trypsin in green, and Bulk_with trypsin in blue; Figure S4: Principal component analysis (PCA) performed on the proteins obtained through spectral count data from the secretome of *Bt407* Cry- and *Bt* Δ*fliK*. PCA is represented by axes 1–2. *Bt407* Cry-_SN in red and *Bt* Δ*fliK*_SN in blue; Figure S5: Principal component analysis (PCA) performed on the proteins obtained by XIC data from the supernatants of *Bt407* Cry- and *Bt* Δ*fliK*. PCA is represented by axes 1–2. *Bt407* Cry-_SN in green, *Bt* Δ*fliK*_SN in blue, and Bulk_SN in red; Table S1: Evaluation of bacterial lysis by counting the number of CFU. Comparable CFU number of biological triplicates for the reference strain *Bt407* Cry-(A) and the mutant strain *Bt* Δ*fliK* (B) with or without treatment with 1 μg of trypsin for 5 min at 37 °C; Table S2: Global representation of the number of identified groups, subgroups, proteins, and peptides in each sample. Groups correspond to proteins with at least one peptide in common, and subgroups correspond to proteins with the same set of identified peptides.
