# Supplementary figures and images for: High-Throughput Analysis of the Flagella FliK-Dependent Surfaceome and Secretome in Bacillus thuringiensis
Source: Biology (Basel). 2025 May 9;14(5):525. doi: 10.3390/biology14050525 (PMC12109265; doi:10.3390/biology14050525)

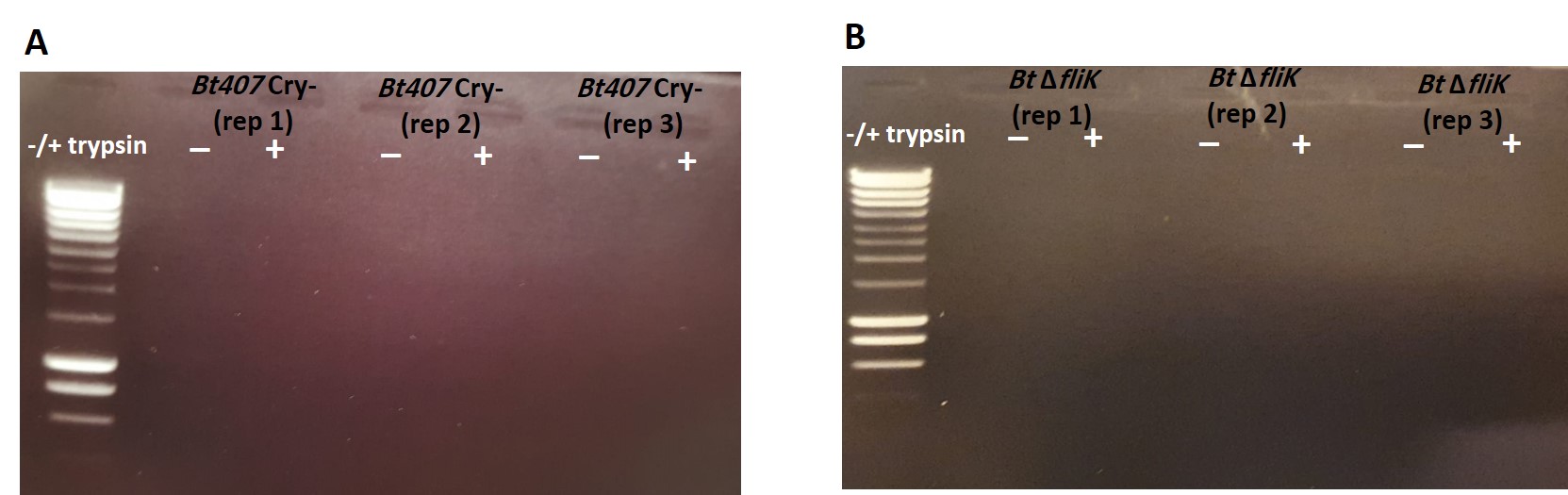

Supplement: Supplementary file 1 [file biology-14-00525-s001.zip › Supplementary Figure S1.jpg]

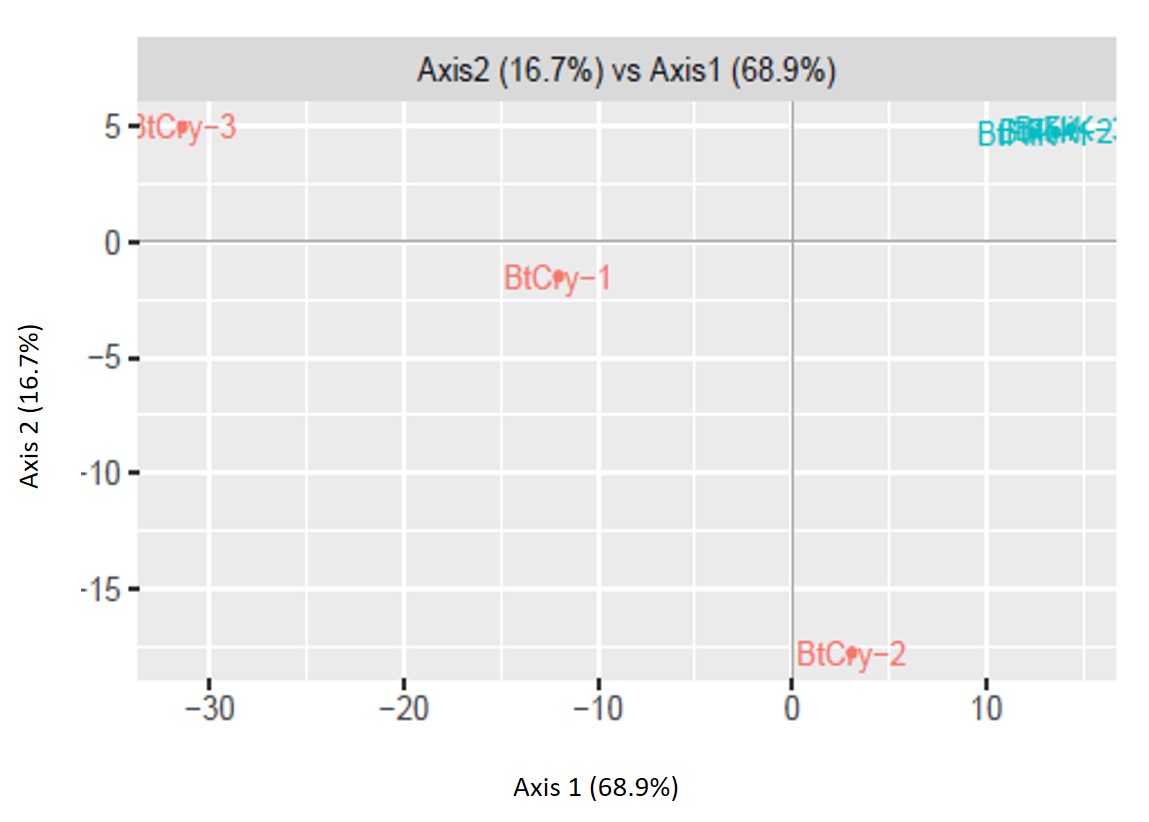

Supplement: Supplementary file 1 [file biology-14-00525-s001.zip › Supplementary Figure S2.jpg]

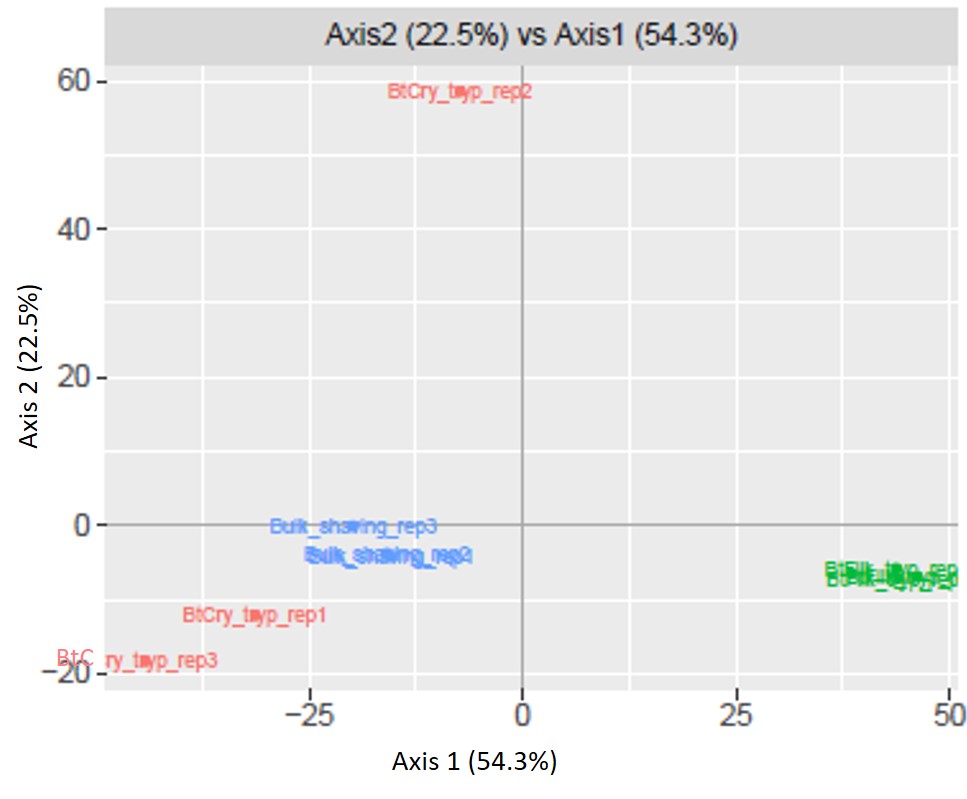

Supplement: Supplementary file 1 [file biology-14-00525-s001.zip › Supplementary Figure S3.jpg]

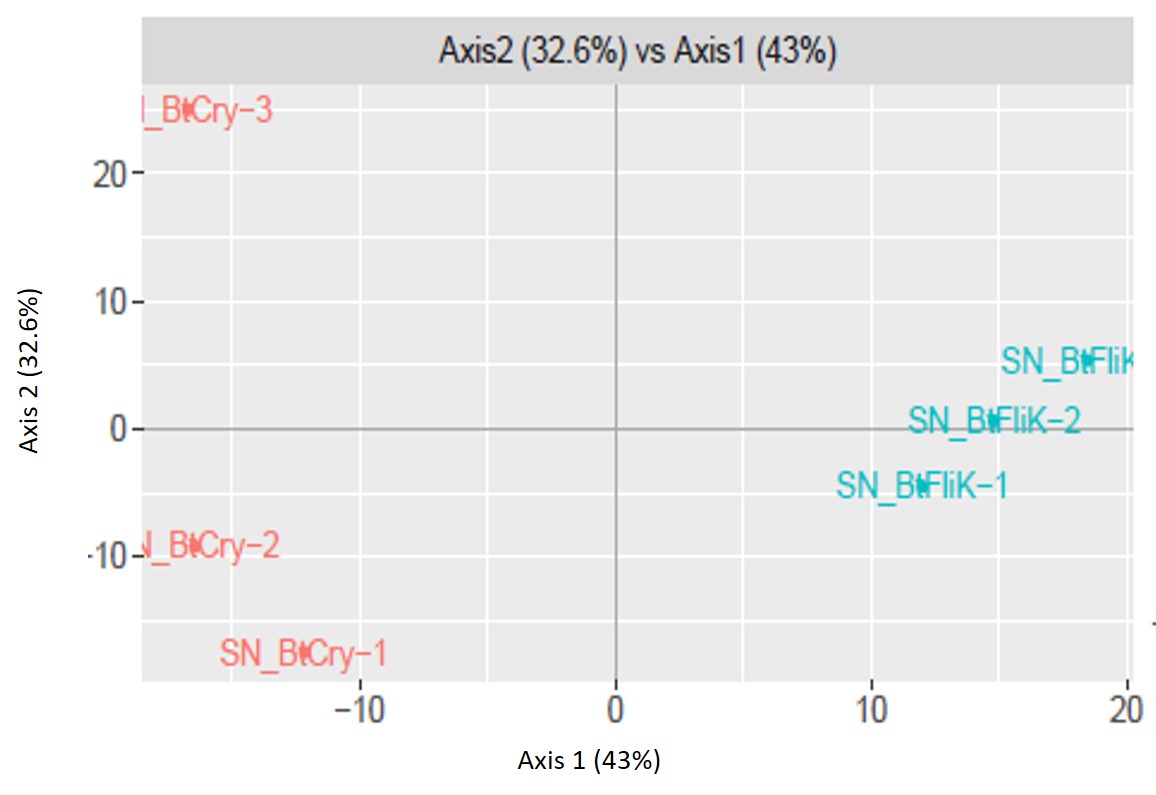

Supplement: Supplementary file 1 [file biology-14-00525-s001.zip › Supplementary Figure S4.jpg]

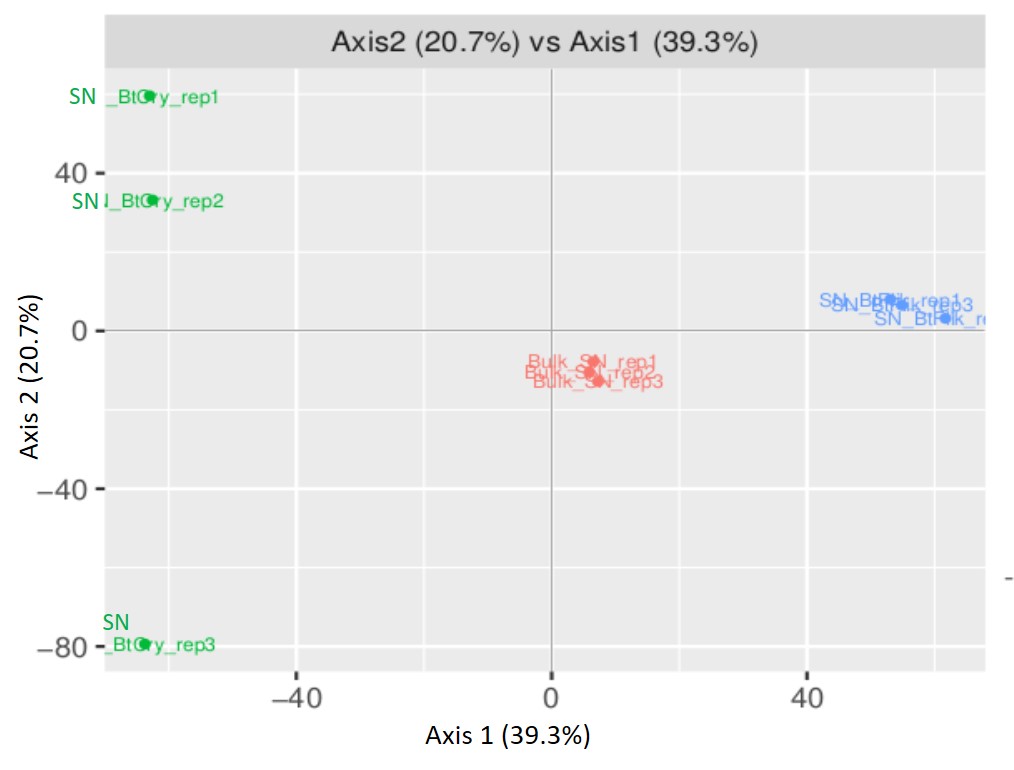

Supplement: Supplementary file 1 [file biology-14-00525-s001.zip › Supplementary Figure S5.jpg]

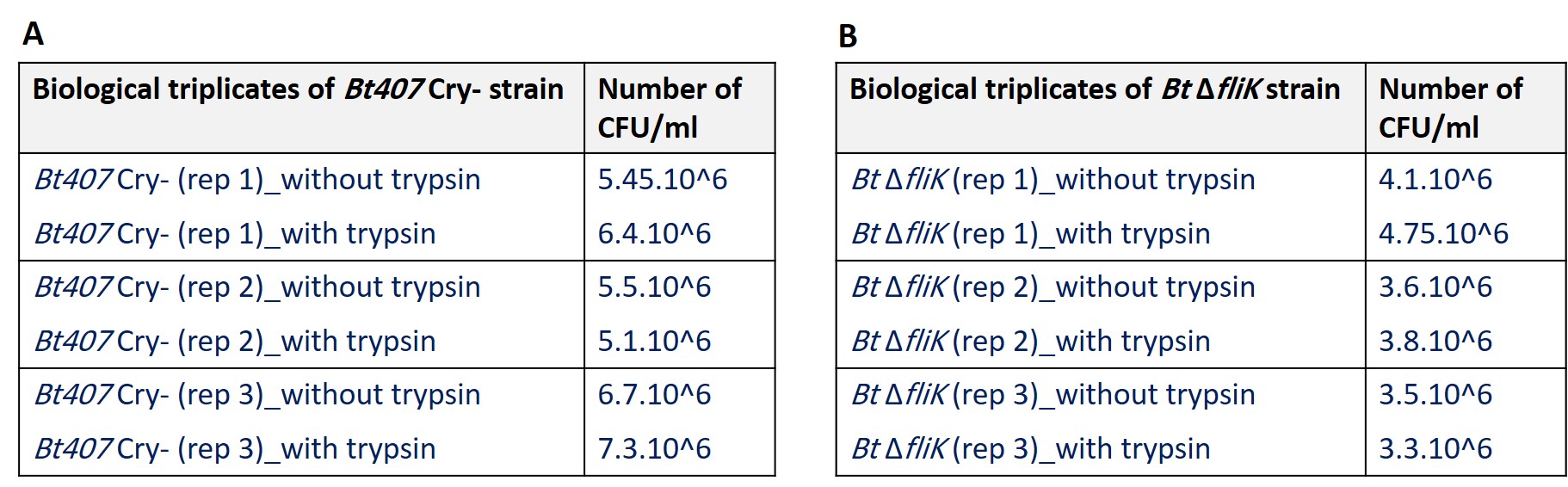

Supplement: Supplementary file 1 [file biology-14-00525-s001.zip › Supplementary Table S1.jpg]

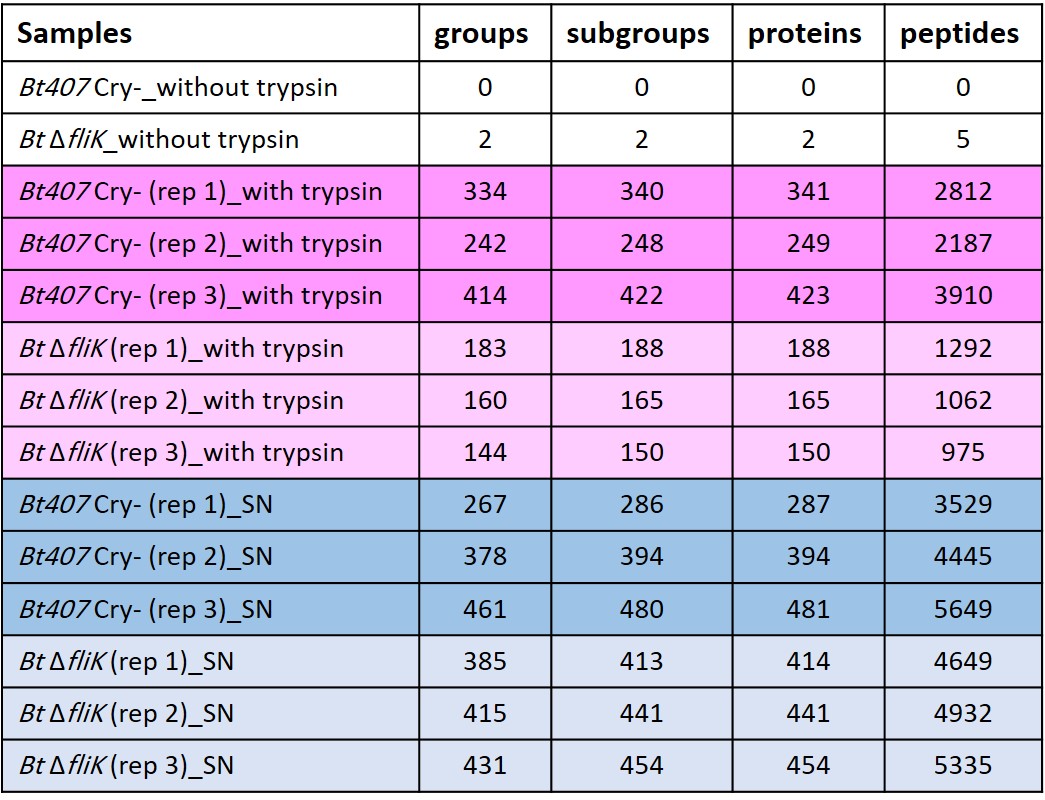

Supplement: Supplementary file 1 [file biology-14-00525-s001.zip › Supplementary Table S2.jpg]
